# JMJD5 inhibits lung cancer progression by facilitating EGFR proteasomal degradation

**DOI:** 10.1038/s41419-023-06194-0

**Published:** 2023-10-09

**Authors:** Jing Shen, Guiling Liu, Hongyan Qi, Xueping Xiang, Jimin Shao

**Affiliations:** 1 Department of Pathology and Pathophysiology, and Department of Medical Oncology of the Second Affiliated Hospital, Zhejiang University School of Medicine, Hangzhou, 310058 China; 2 Experimental Teaching Center of Basic Medicine, Zhejiang University School of Medicine, Hangzhou, 310058 China; 3 Department of Pathology, the Second Affiliated Hospital, Zhejiang University School of Medicine, Hangzhou, 310058 China; 4 Department of Pathology and Pathophysiology, and Cancer Institute of the Second Affiliated Hospital, Zhejiang University School of Medicine, Hangzhou, 310058 China

**Keywords:** Cancer, Cancer

## Abstract

Aberrant activation of epidermal growth factor receptor (EGFR) signaling is closely related to the development of non-small cell lung cancer (NSCLC). However, targeted EGFR therapeutics such as tyrosine kinase inhibitors (TKIs) face the challenge of EGFR mutation-mediated resistance. Here, we showed that the reduced JmjC domain-containing 5 (JMJD5) expression is negatively associated with EGFR stability and NSCLC progression. Mechanically, JMJD5 cooperated with E3 ligase HUWE1 to destabilize EGFR and EGFR TKI-resistant mutants for proteasomal degradation, thereby inhibiting NSCLC growth and promoting TKI sensitivity. Furthermore, we identified that JMJD5 can be transported into recipient cells via extracellular vesicles, thereby inhibiting the growth of NSCLC. Together, our findings demonstrate the tumor-suppressive role of JMJD5 in NSCLC and suggest a putative therapeutic strategy for EGFR-related NSCLC by targeting JMJD5 to destabilize EGFR.

## Introduction

Epidermal growth factor receptor (EGFR) enacts critical roles in epithelial tissue maintenance and is often abnormally activated in epithelial malignancies, including lung cancer [1–3]. EGFR signaling activation, such as gene amplification and somatic activating mutations (e.g., L858R and exon 19 deletions), is a major driver of non-small-cell lung cancer (NSCLC), which constitutes more than 85% of all lung cancer cases [3, 4]. Currently, molecular targeted tyrosine kinase inhibitors (TKIs), such as gefitinib and erlotinib, have been widely used to treat patients with EGFR-activating mutations in NSCLC and have significantly improved patient outcomes [5]. Unfortunately, despite the well initial clinical responsiveness, most patients inevitably developed acquired resistance to EGFR-TKIs, in which the gatekeeper mutation T790M of EGFR accounts for about 50% of all TKIs resistant cases. Although new generation TKIs (e.g., osimertinib) have been developed to overcome T790M-driven resistance, newly acquired EGFR-resistant mutations have also emerged (e.g., EGFR C797S and G796D/R/S mutations) [6]. Furthermore, in addition to the occurred resistance, EGFR TKIs are more associated with EGFR-activating mutations than wild-type EGFR (WT-EGFR), indicating that this strategy was of relatively little benefit to the majority of lung cancer patients with WT-EGFR. Increasing evidence suggests that elevated WT-EGFR expression not only contributes to the pathogenesis of lung cancer but also associates with acquired EGFR TKIs resistance [6–9]. Thus, it is necessary to discover new reliable therapeutic strategies targeting either mutant EGFR or WT-EGFR with alternative mechanisms.

The binding of ligands to the extracellular domain of EGFR initiates receptor dimerization, phosphorylation, and downstream signaling activation (MAPK, PI3K/AKT, JAK/STAT, etc.), which controls cell growth and survival [10]. Internalized EGFR can be degraded or recycled back to the cell surface, which is crucial for signal termination or maintenance. Accumulating studies show that dysregulation of EGFR spatial distribution and stability also critically contributes to abnormal EGFR signaling and lung cancer progression [3]. Mitogen-inducible gene 6 (Mig6) fails to mediate the degradation of mutant EGFR, and deletion of Mig6 promotes tumor initiation and mutant EGFR-driven lung cancer progression [11]. Pseudokinase Tribble 3 (TRIB3) contributes to NSCLC development by increasing EGFR recycling and stability [12]. Activation of F-box protein FBXL2 destabilizes EGFR, thereby inhibiting EGFR-driven NSCLC growth [13]. These findings highlight promoting EGFR degradation as an alternative strategy against EGFR-related cancers.

JmjC (Jumonji-C) domain-containing 5 (JMJD5) (also called KDM8) is a 2-oxoglutarate (2-OG) dependent oxygenase of the JmjC subfamily. Although JMJD5 was originally defined as a lysine demethylase [14], we and others later identified its role as a hydroxylase and protease [15–18]. By shuttling between the nucleus and cytoplasm, JMJD5 could either associate with histones or cytosolic proteins, and thus plays an important role in embryonic development [19, 20], circadian regulation [21, 22], osteoclastogenesis [15], and stem-cell renewal [23]. JMJD5 influences gene transcription through a variety of mechanisms, including regulation of protein stability, nuclear translocation of transcription factors, and digestion of methylated histone tails to modulate chromatin conformation [15, 17, 18, 24–26]. Recently, JMJD5 has also been discovered to associate with cancer development. It is highly expressed in breast, prostate, and colorectal tumors as well as oral squamous cell carcinomas and promotes the growth of respective cancer cell lines [14, 25, 27–30]. On the other hand, JMJD5 is downregulated in hepatocellular carcinoma [31] and pancreatic cancer [32] and negatively regulates cancer progression, indicating heterogeneity of JMJD5 among different tumor types. Notably, our previous study found that JMJD5 is downregulated in malignant effusions and lung cancer tissues of patients [33]. However, the role and underlying mechanisms of JMJD5 in lung cancer remain unclear.

In this study, we identified that the reduced JMJD5 expression is negatively associated with EGFR expression and signal activation, and correlated with better survival of NSCLC. We further demonstrated that JMJD5 interacts with EGFR and promotes its ubiquitination and proteasomal degradation by recruiting E3 ligase HUWE1. Notably, overexpression of JMJD5 significantly inhibits NSCLC cell growth and promotes the sensitivity of cells to EGFR TKI, and enhanced exosomal transferring of JMJD5 may provide a novel therapeutic strategy for NSCLC to target EGFR.

## Materials and methods

### Cell culture and reagents

Human NSCLC cell lines A549, H1299, H1975, H1650, HCC827, PC9, and H226 and human leukemia K562 cells were purchased from the Cell Bank of the Chinese Academy of Sciences (Shanghai, China) and cultured in RPMI 1640 medium (Invitrogen, USA). All cells were cultured at 37 °C and 5% CO_2_. All cell lines have been validated by STR profiling within the last three years and were tested for mycoplasma and microbial contamination.

Recombinant human EGF was purchased from PeproTech (PeproTech, USA). MG132 (S2619), Gefitinib (S1025), and AZD9291 (S7297) were from Selleck (Selleck, USA). Cycloheximide (HY-12320) and Chloroquine (HY-17589A) were from MedChemExpress (MCE, USA).

### Plasmids, transfection, and lentiviral infection

The full-length and deletion mutants of human EGFR and JMJD5 were constructed into a pcDNA3.1(+) vector with a 6×Myc-tag or a Flag-tag. Human pENTR1A-HUWE1 was obtained from addgene (#37431). A pLVX-puro vector was used to generate recombinant lentiviruses expressing human JMJD5 with a Flag-tag or human EGFR with an eGFP-tag. Lentiviral plasmids and packaging plasmids (psPAX2, pMD2.G) were co-transfected into HEK293FT cells to produce lentivirus. The short hairpin RNA (shRNA) targeting human JMJD5 was generated by the insertion of specific oligos into a pLVX shRNA1 lentiviral vector. The transfection of siRNAs targeting human JMJD5, EGFR, and HUWE1 were performed by using Lipofectamine™ RNAiMAX (Invitrogen, USA) according to the manufacturer’s instruction. Sequences of shRNA and siRNA were shown in Table S1. Primers were shown in Table S2.

### Western blotting and co-immunoprecipitation (Co-IP)

Western blotting and Co-IP were performed as previously described [34]. In this study, the following antibodies were used: EGFR (1902-1) was from Epitomics (Epitomics, USA). JMJD5 (ab36104) was from Abcam (Abcam, UK). EGFR (sc-373746), JMJD5 (sc-377440), Ubiquitin (sc-8017), and Myc (sc-789) were from Santa Cruz Biotechnology (Santa Cruz, USA). p-EGFR (#3777), p-AKT (#9271), AKT (#2920), p-ERK (#4370), ERK (#4696), EEA1 (#3288), LAMP1 (#9091), and Flag (#14793) were from Cell Signaling Technology (CST, USA). HUWE1 (A700-129) was from Bethyl (Bethyl, USA). RAB11 (15903-1-AP) was from Proteintech (Proteintech, China). GAPDH (R1201-1) was from HuaBio (HuaBio, China). Original western blots were presented in Supplementary File.

### RNA-seq analysis

Total cellular RNA was extracted by using TRIzol reagent (Invitrogen, USA) and RNA-seq analysis was carried out by Biomarker Technologies (Biomarker, China). Mapped gene counts were determined using HT Seq v0.6.1. RPKM (Reads Per Kilo bases per Million reads) was calculated according to the length of the gene and the reads count mapped to the gene. Differential genes were identified with corrected q-value less than 0.05 and fold change greater than 2.

### EGFR degradation assay

For degradation with EGF stimulation, cells were maintained in a serum-free medium overnight and treated with EGF (100 ng/ml) for the different indicated times. For degradation without EGF stimulation, cells were maintained in a serum-free medium containing 100 μg/ml cycloheximide (CHX) for the different indicated times. Cells were then harvested and lysed, and proteins were analyzed by Western blotting.

### Ubiquitination assays

For the EGFR ubiquitination assay, HEK293T cells were co-transfected with EGFR-Flag, HA-ubiquitin-Lys 48, HUWE1, Myc-JMJD5, or empty vector for 48 h. Then cells were treated with MG132 (10 μM) for 6 h before harvesting. Immunoprecipitation was performed using anti-FLAG M2 Magnetic Beads (Sigma-Aldrich, USA) and followed by Western blotting analysis.

### Exosome isolation, identification, and uptake

After incubating with exosome free medium for 72 h, the culture supernatants of H1975 cells were collected. Cell debris and large vesicles were removed by centrifugation (500 rcf for 15 min, 2000 rcf for 20 min, and 10,000 rcf for 40 min). The supernatants were further ultracentrifuged at 100,000 rcf for 90 min and isolated exosomes were collected and resuspended in PBS. All steps were performed at 4 °C. Exosomes stained with 2% uranyl acetate were visualized under transmission electron microscopy (Tecnai G2 Spirit, FEI, Czech Republic) at 80.0 kV. A Zeta View system (Particle Metrix, Germany) was used to analyze the size and number of exosomes. For exosome uptake, cells seeded on coverslips were incubated with PKH67 (MKCK7852, Sigma) labeled exosomes for 4–12 h and fixed with 4% paraformaldehyde. The cell membrane was stained with CM-DiI (C7000, Thermo) for 15 min. Cells were then permeabilized with 0.2% Triton-100 and the nuclei were stained with DAPI.

### Colony formation assay

500–1000 cells per well were seeded into the 6-well dish and cultured with exosome-containing medium (30 μg/ml) or not for about 10 days. After fixing with 4% paraformaldehyde, cells were stained with crystal violet. Colony numbers were counted under the microscope and one colony that more than 50 cells was counted.

### Transwell assay

4–6 × 10^4^ cells were seeded in the upper chamber of the transwell apparatus (Millipore, USA) containing serum-free medium, and a complete medium containing 30 μg/ml exosome or not was added to the bottom chamber. After 24 h incubation, the migrated cells were further fixed with 4% paraformaldehyde and stained with crystal violet.

### Xenograft model

The Laboratory Animals Welfare Ethics Review Committee of Zhejiang University approved all animal studies. All mice were randomized into different groups after age and sex matching. 5 × 10^6^ cells were injected subcutaneously into BALB/C nude mice (4-week-old, male) (SLAC, China) (*n* = 5/group). When the tumor volume was about 50–100 mm^3^, mice were administered with gefitinib (20 mg/kg, intraperitoneal injection) or AZD9291 (5 mg/kg, intragastric administration) once every two days or exosomes (100 μg/mouse, intravenous injection) once a week for 2 weeks. No blinding method was used during administration. The size of xenograft tumor was measured and calculated according to the formula: 0.5 × length × width^2^. The mice were sacrificed after 4 weeks and xenograft tumors were harvested, weighed, and photographed.

### Immunohistochemistry (IHC) staining

The LUAD tumor specimens (*n* = 108) in this study were obtained from the Second Affiliated Hospital of Zhejiang University School of Medicine. The ethics committee of the Zhejiang University School of Medicine approved the study. IHC was carried out using Envision Detection System (DAKO, Carpinteria, CA) with anti-EGFR (sc-373746) and anti-JMJD5 (sc-377440). Two independent investigators blinded to the clinical data assessed and confirmed the staining results.

### Statistical analysis

All results in this paper were performed at least three independent repeats and data were presented as mean ± SD. Statistical analysis was performed by Student’s t-tests and one-way ANOVA. The survival curves were analyzed using the Kaplan–Meier method. *P*-value < 0.05 was considered statistically significant.

## Results

### JMJD5 is negatively related to EGFR expression and correlated with better survival of lung cancer

**Fig. 1:**
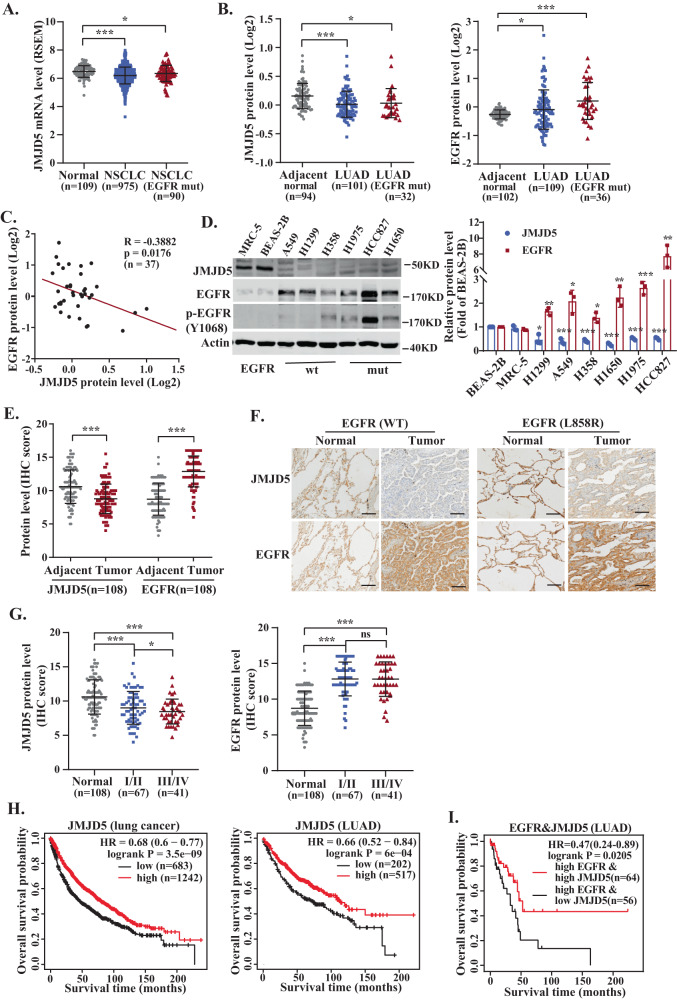
JMJD5 is negatively related to EGFR expression and correlated with better survival of lung cancer. **A** TCGA database analysis of JMJD5 mRNA expression in NSCLC, NSCLC with EGFR mutation, and normal tissues. **B** CPTAC database analysis of JMJD5 and EGFR protein expression in LUAD, LUAD with EGFR mutation, and adjacent normal tissues. **C** Correlation analysis of JMJD5 and EGFR protein expression in NSCLC tissues (adenocarcinoma and squamous cell carcinoma) with EGFR mutations from CPTAC database. **D** Western blot analysis of JMJD5 and EGFR protein expression in human bronchial epithelial cells (BEAS-2B), human embryonic lung fibroblasts (MRC-5) and NSCLC cells (A549, H1299, H358, H1975, HCC827, and H1650) (*n* = 3). IHC analysis of JMJD5 and EGFR protein expression in LUAD patient tissues and adjacent normal tissues (**E**, **F**), and in LUAD patient tissues with different stages (**G**). Scale bar: 100 μm. Kaplan–Meier plots of overall survival of human lung cancer patients were stratified by JMJD5 levels from the Kaplan–Meier plotter database (**H**) or JMJD5 and EGFR coexpression levels from the cBioPortal database (**I**). ns no significant, *P < 0.05, **P < 0.01, ***P < 0.001.

By analyzing The Cancer Genome Atlas (TCGA) and Clinical Proteomic Tumor Analysis Consortium (CPTAC) databases, we found that the mRNA (Fig. 1A) and protein (Fig. 1B) level of JMJD5 was significantly decreased in NSCLC tissues compared with the normal tissues. To clarify the functions and regulatory molecules of JMJD5 in cancer, we analyzed JMJD5-associated proteins using mass spectrometry-based proteomic analysis. Interestingly, EGFR was successfully identified as a major partner of JMJD5 (Fig. S1A). Notably, JMJD5 also exhibited a significant reduction level in EGFR mutant NSCLC tissues, and a negative correlation could be found between JMJD5 and EGFR protein levels (Fig. 1A–C). We then investigated the relationship between JMJD5 and EGFR expression. There was no correlation between the mRNA levels of JMJD5 and EGFR, and the mRNA level of EGFR did not exhibit significant changes after the overexpression of JMJD5 (Figs. S1B–D). However, low JMJD5 protein expression was associated with increased EGFR protein expression in most human NSCLC cell lines, either wild-type or mutant EGFR (Fig. 1D). Moreover, IHC analysis of 108 clinical lung cancer tissues revealed a significant decrease of JMJD5 and increase of EGFR level in tumors compared to adjacent tissues (Fig. 1E, F). We further analyzed the expression of JMJD5 at different stages of human lung adenocarcinoma (LUAD). As shown in Fig. 1G and Fig. S1E, IHC and TCGA analyses showed that JMJD5 expression was declined during LUAD development. Survival analysis showed that high expression levels of JMJD5 were positively correlated with better overall survival and post-progression survival of lung cancer patients (Figs. 1H and S1F, G). Furthermore, in NSCLC patients with high EGFR expression, those with high JMJD5 expression have a better survival rate than those with low JMJD5 expression (Fig. 1I).

### JMJD5 downregulates the protein level of EGFR and inhibits its signaling activity

**Fig. 2:**
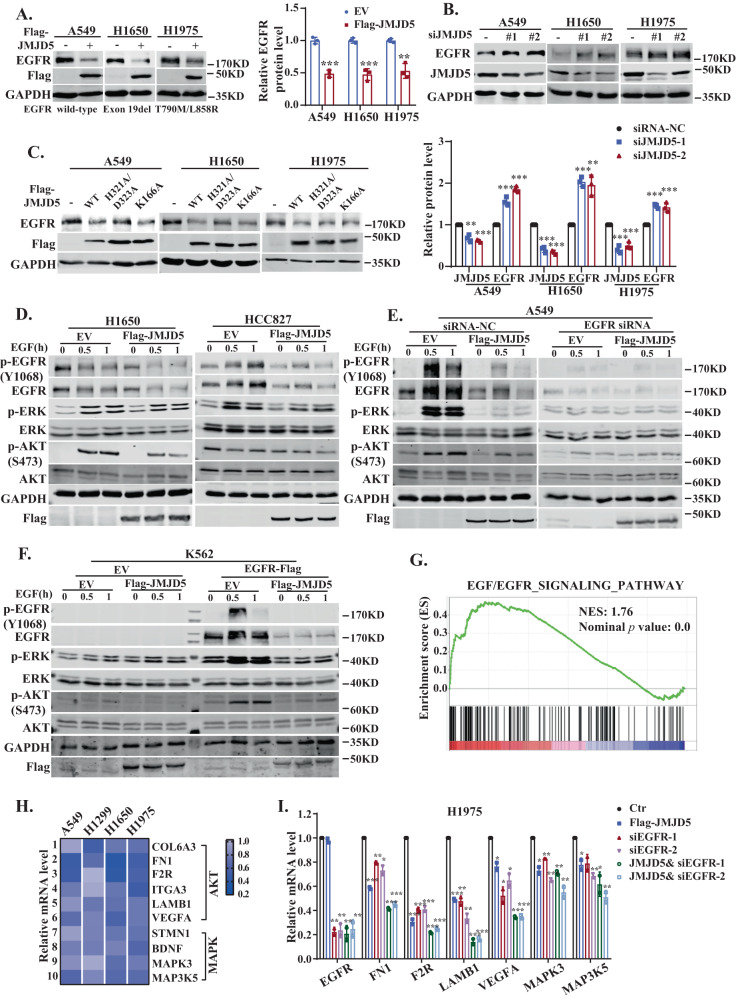
JMJD5 downregulates the protein level of EGFR and inhibits its signaling activity. Western blot analysis of EGFR protein levels in NSCLC cells after JMJD5 overexpression (**A**) or siRNA knockdown (**B**). **C** Western blot analysis of EGFR protein levels after ectopically expressing JMJD5 wild-type (WT) or mutants (H321A/D323A or K166A). **D**–**F** Cancer cells were stimulated with or without EGF (100 ng/ml). Indicated proteins were analyzed by Western blot analysis. **G** Gene set enrichment plots of downregulated genes belonging to the EGF-EGFR pathway in JMJD5 stably expressed H1975 cells. NES: normalized enrichment score. **H**, **I** RT-qPCR analysis of the downregulated genes in JMJD5 stably expressed NSCLC cells with or without EGFR silencing. The analyses were repeated three times, and the results were expressed as mean ± SD. *P < 0.05, **P < 0.01, ***P < 0.001.

To elucidate the relationship and underlying mechanisms between JMJD5 and EGFR, we first established JMJD5 stably overexpressed NSCLC cell lines (A549 cells with high expression of WT-EGFR; H1650 and H1975 cells with EGFR E19del or EGFR^L858R/T790M^, respectively) and examined EGFR expression. As shown in Fig. 2A and Fig. S2A, ectopic expression of JMJD5 markedly reduced the protein expression but not the mRNA level of WT-EGFR and mutant EGFR. Conversely, the silencing of JMJD5 led to the upregulation of EGFR proteins without affecting mRNA expression (Figs. 2B and S2B). Meanwhile, we also ectopically expressed JMJD5 mutants in NSCLC cells. JMJD5 nuclear localization signal mutant (K166A), which is unable to enter the nucleus, reduced EGFR protein levels similar to wild-type JMJD5, whereas the catalytically inactive mutant of JMJD5 (H321A/D323A) showed a slightly weaker effect (Figs. 2C and S2C). These results suggest that JMJD5 downregulates EGFR protein expression, which is largely independent of its nuclear localization and enzymatic functions.

Next, we examined whether JMJD5 could regulate EGFR downstream signaling. As shown in Fig. 2D, E, enforced JMJD5 expression suppressed EGF-induced phosphorylation of both WT and mutant EGFR (TKI-resistant cell H1650 and TKI-sensitive cell HCC827) as well as the activation of downstream ERK and AKT signaling. By contrast, silencing EGFR effectively abrogated the suppression effects (Fig. 2E). Moreover, in EGFR-negative human leukemia K562 cells, overexpression of JMJD5 only inhibited the activation of ERK and AKT pathways after exogenous EGFR expression (Fig. 2F). We further analyzed gene expression in H1975 cells with stable overexpression of JMJD5 by RNA sequencing. GSEA analysis showed that in the genes downregulated after JMJD5 overexpression, EGF/EGFR signaling pathway-related genes and downstream genes of PI3K/AKT and MAPK pathways were significantly enriched (Figs. 2G and S2D). The alterations of related genes were confirmed in NSCLC cell lines with WT or mutant EGFR after enforced JMJD5 expression (Fig. 2H). The combination of JMJD5 overexpression and EGFR silencing further decreased the levels of these genes (Fig. 2I). Altogether, our results suggest that JMJD5 downregulates the expression and phosphorylation of EGFR, which further inhibits the activation of EGFR downstream signaling pathways.

### JMJD5 relies on its JmjC domain to interact with the TK domain of EGFR

**Fig. 3:**
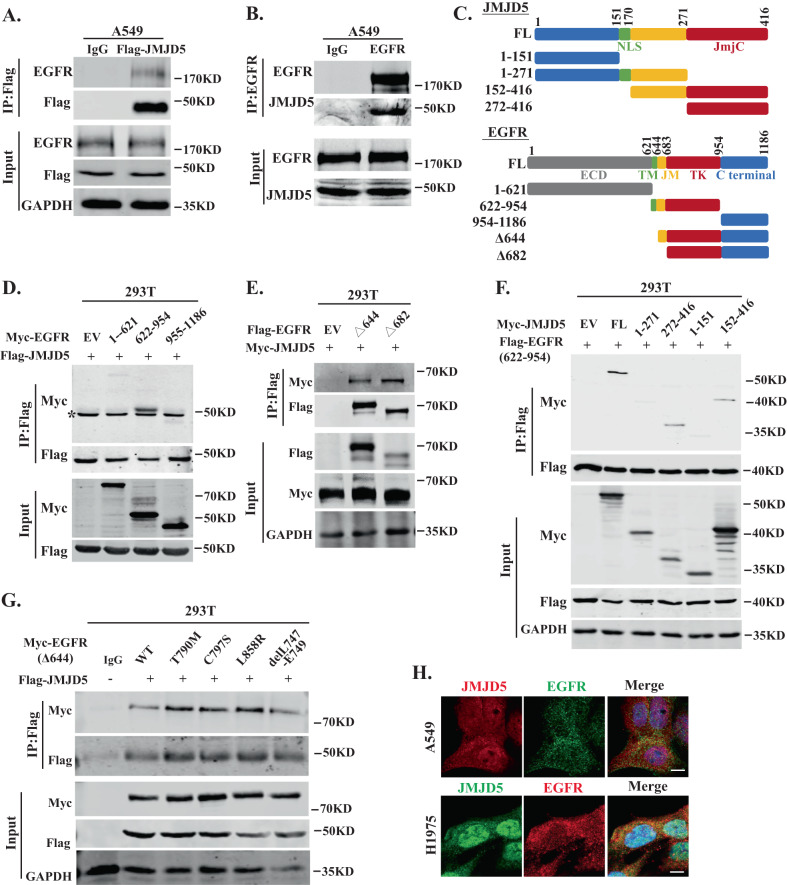
JMJD5 relies on its JmjC domain to interact with the TK domain of EGFR. After anti-Flag (**A**) or anti-EGFR (**B**) immunoprecipitation, coprecipitated proteins were detected by indicated antibodies in A549 cells. **C** Schematic representation of the Myc- or Flag-tagged JMJD5 and EGFR fragment constructs. FL represents full-length JMJD5 or EGFR. **D**–**F** Co-IP analysis of the interaction regions between EGFR and JMJD5. **G** Co-IP analysis of the association between JMJD5 and EGFR mutants. **H** Colocalization of JMJD5 and EGFR was analyzed with confocal microscopy. The scale bar represents 7.5 μm. The analyses were repeated three times.

As suggested by the mass spectrometry data in Fig. S1A, JMJD5 probably influences the function of EGFR by interacting with it. To confirm this point, we performed co-immunoprecipitation (Co-IP) assay to examine the association between JMJD5 and EGFR. Co-IP analysis revealed that EGFR interacted with exogenous and endogenous JMJD5 (Fig. 3A, B). We further constructed different deletion mutants of JMJD5 and EGFR to map the interaction regions of the two proteins (Fig. 3C). The tyrosine kinase (TK) domain, rather than the adjacent intracellular juxtamembrane (JM) domain, was identified to interact with JMJD5 (Fig. 3D, E), whereas the JmjC domain (amino acids 272–416) of JMJD5 was responsible for the association between JMJD5 and EGFR (Fig. 3F). We then examined the interaction between JMJD5 and common EGFR mutants in NSCLC patients that drive tumorigenesis. Interestingly, all EGFR mutants were able to bind JMJD5, with the TKI-resistant mutants (T790M, C797S) and activating mutant L858R of EGFR showing even stronger binding abilities than WT-EGFR (Fig. 3G). In addition, the immunofluorescence assay showed that JMJD5 was mainly colocalized with WT or mutant EGFR in the cell membrane and cytoplasm (Fig. 3H).

### JMJD5 promotes the proteasomal degradation of EGFR by recruiting E3 ligase HUWE1

**Fig. 4:**
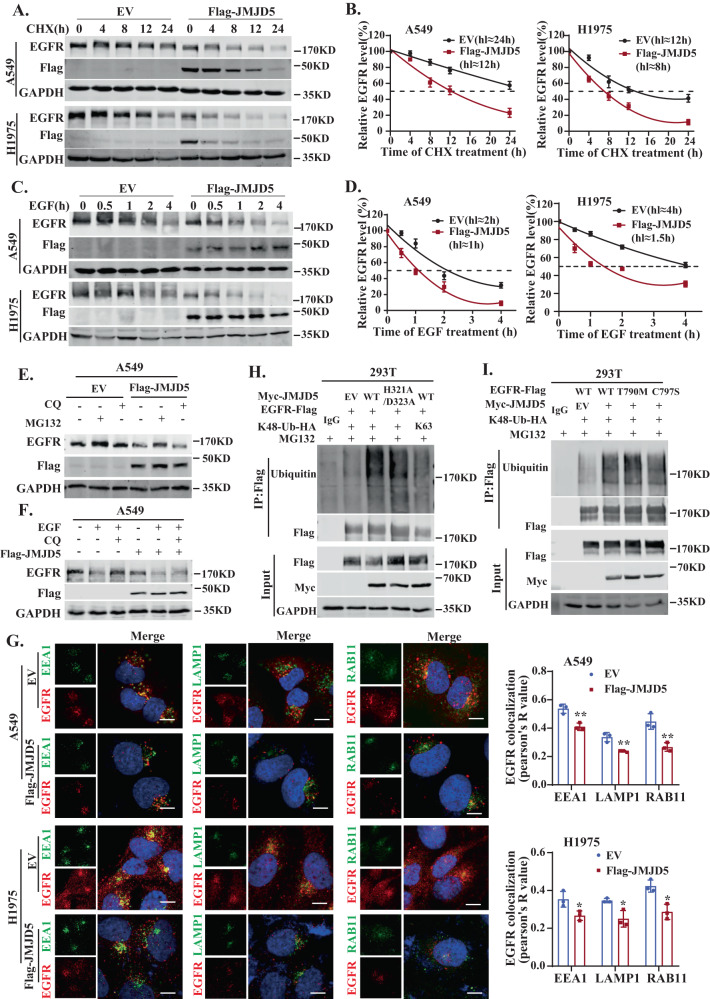
JMJD5 promotes the proteasomal degradation of EGFR. **A**, **B** Control or JMJD5 stably expressed A549 or H1975 cells were treated with cycloheximide (CHX) (100 μg/ml) at indicated intervals and protein stability of EGFR was analyzed by Western blot. **C**, **D** Western blot analysis of EGFR degradation rates after EGF stimulation in A549 or H1975 cells. **E**, **F** A549 cells stably expressing JMJD5 were treated with 10 μM MG132 for 6 h or 20 μM CQ for 6 h before Western blot analyses. **G** Colocalization analysis of EGFR with EEA1, Rab11, and LAMP-1 after 30 min EGF treatment (100 ng/ml) in control and JMJD5 overexpressing A549 or H1975 cells. The scale bar represents 7.5 μm. Quantification of EGFR/EEA1, EGFR/Rab11, and EGFR/LAMP-1 colocalization was shown as Pearson’s coefficient. **H** IP-Western analysis of EGFR K48- or K63-ubiquitination after ectopically expressing JMJD5 wild-type (WT) or mutant (H321A/D323A). **I** IP-Western analysis of the K48-ubiquitination of EGFR wild-type (WT) or mutants (T790M, C797S) after JMJD5 overexpression. The analyses were repeated three times, and the results were expressed as mean ± SD. *P < 0.05, **P < 0.01, ***P < 0.001.

Dysregulation of EGFR protein stability greatly contributes to aberrant EGFR signaling and lung cancer progression [3]. To determine whether JMJD5 reduces EGFR protein level by facilitating its degradation, we examined differences in the stability of EGFR protein after changes in JMJD5 expression. Overexpression of JMJD5 in A549 (harboring WT-EGFR) or H1975 (harboring L858R/T790M double mutations) cells decreased the half-life of EGFR with or without EGF induction (Fig. 4A–D). By contrast, depletion of JMJD5 prolonged the half-life of EGFR (Fig. S3A, B). To further clarify whether JMJD5 affects EGFR stability through proteasome or lysosome pathway, we treated cells with proteasome inhibitor MG132 or lysosome inhibitor chloroquine (CQ). Consistent with prior reports [13, 35, 36], in the absence of EGF induction, the degradation of EGFR protein proceeds primarily through the proteasome pathway, while following EGF stimulation, the EGFR protein was rapidly destabilized mainly through the lysosome pathway (Fig. S3C, D). The decrease of EGFR level caused by JMJD5 overexpression can be effectively reversed by MG132 but not by CQ, suggesting that proteasome-mediated degradation is the primary cause of JMJD5-induced EGFR level decline (Figs. 4E, F, S3E). Accordingly, overexpression of JMJD5 reduced EGFR colocalization with the early endosomal marker EEA-1, the recycling endosome marker RAB11, and the lysosomal degradation marker LAMP-1, indicating that JMJD5 downregulated EGFR expression, thereby compromising EGF-induced EGFR endocytosis and subsequent intracellular sorting (Fig. 4G). The reduced colocalization of EGFR with EEA1 after JMJD5 overexpression was rescued in the presence of MG132 (Fig. S3F). Moreover, ectopic expression of wild-type JMJD5 and its catalytically inactive mutant remarkably increased Lys 48 (K48)-linked, but not Lys 63-linked, polyubiquitin chains of EGFR protein (Fig. 4H). Notably, JMJD5 also increased K48-linked ubiquitination of EGFR TKI-resistant mutants (T790M、C797S) (Fig. 4I). These results suggest that JMJD5 promotes EGFR polyubiquitination, resulting in proteasome-mediated EGFR degradation, both in WT-EGFR and its resistant mutants.

**Fig. 5:**
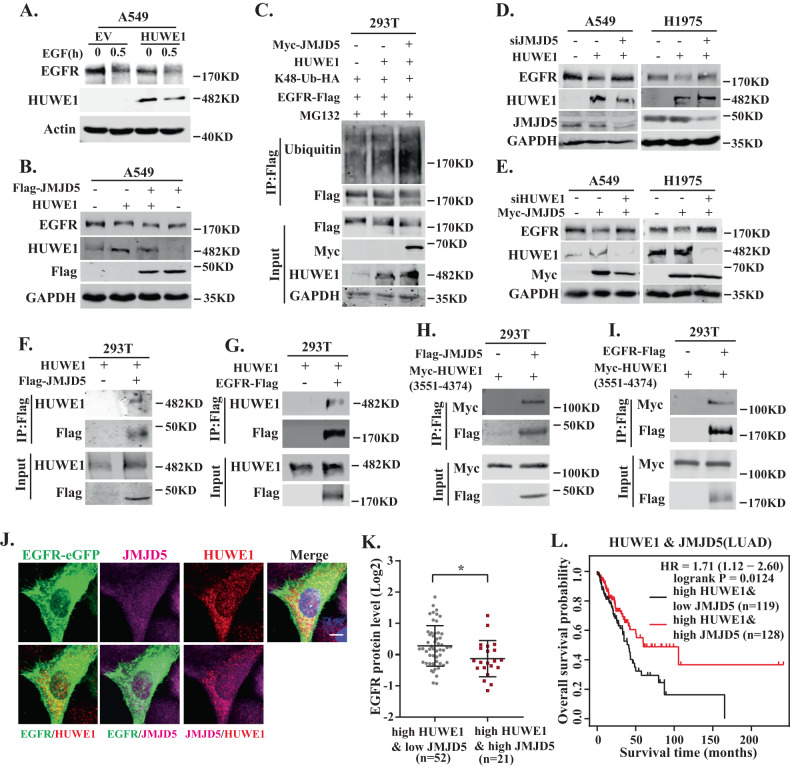
JMJD5 promotes EGFR degradation by recruiting E3 ligase HUWE1. Western blot analysis of EGFR protein levels after HUWE1 overexpression with or without EGF stimulation (**A**) or coexpression of HUWE1 and JMJD5 (**B**). **C** IP-Western analysis of EGFR K48-ubiquitination after coexpression of HUWE1 and JMJD5. HUWE1 (**D**) or JMJD5 overexpressing (**E**) cells were simultaneously treated with JMJD5 siRNA or HUWE1 siRNA, and EGFR protein levels were analyzed by Western blot. **F**–**I** After anti-Flag immunoprecipitation, coprecipitated proteins were detected by indicated antibodies in 293T cells. **J** Colocalization of JMJD5, EGFR-eGFP, and HUWE1 was analyzed with confocal microscopy in H1975 cells. The scale bar represents 7.5 μm. CPTAC database analysis of EGFR protein levels (**K**) or TCGA database analysis of overall survival (**L**) of LUAD patients with high HUWE1 expression combined with high or low JMJD5 expression. The analyses were repeated three times. *P < 0.05.

It has been reported that JMJD5 facilitates the proteasomal degradation of binding proteins by promoting its association with E3 ubiquitin ligase [15, 24]. Interestingly, when we screened the interacting proteins of JMJD5, the HECT-domain E3 ligase HUWE1 was successfully identified (Fig. S1A). Overexpression of HUWE1 decreased EGFR protein levels either in the presence or absence of EGF in A549 cells (Fig. 5A, B). Ectopic expression of HUWE1 elevated K48-linked ubiquitination of EGFR (Fig. 5C). Notably, coexpression of JMJD5 with HUWE1 further downregulated EGFR protein level and increased EGFR ubiquitination (Fig. 5B, C). Next, we investigated the role of JMJD5 and HUWE1 in EGFR destabilization. As shown in Fig. 5D, E, depletion of JMJD5 in A549 and H1975 cells restored HUWE1-medicated EGFR degradation, whereas silencing HUWE1 rescued JMJD5-enhanced EGFR downregulation, indicating that both JMJD5 and HUWE1 are required to modulate EGFR stability. Co-IP analysis showed that both the full-length and the C-terminus (3551-4370 truncate containing HECT domain) of HUWE interacted with JMJD5 and EGFR (Fig. 5F–I). Immunofluorescence analysis also revealed that there was a strong colocalization of JMJD5, HUWE1, and EGFR in cancer cells (Fig. 5J). We then detected the expression of HUWE1 in human NSCLC. Intriguingly, the mRNA (Fig. S4A) and protein (Fig. S4B) level of HUWE1 was significantly elevated in NSCLC tissues compared with the normal tissues. However, NSCLC patients with high expression of HUWE1 and JMJD5 had significantly lower EGFR protein levels than those with low JMJD5 expression (Fig. 5K), as well as correlated with a better survival rate of the disease (Fig. 5L).

### JMJD5 inhibits cell proliferation, migration and promotes gefitinib-sensitivity in lung cancer cells

**Fig. 6:**
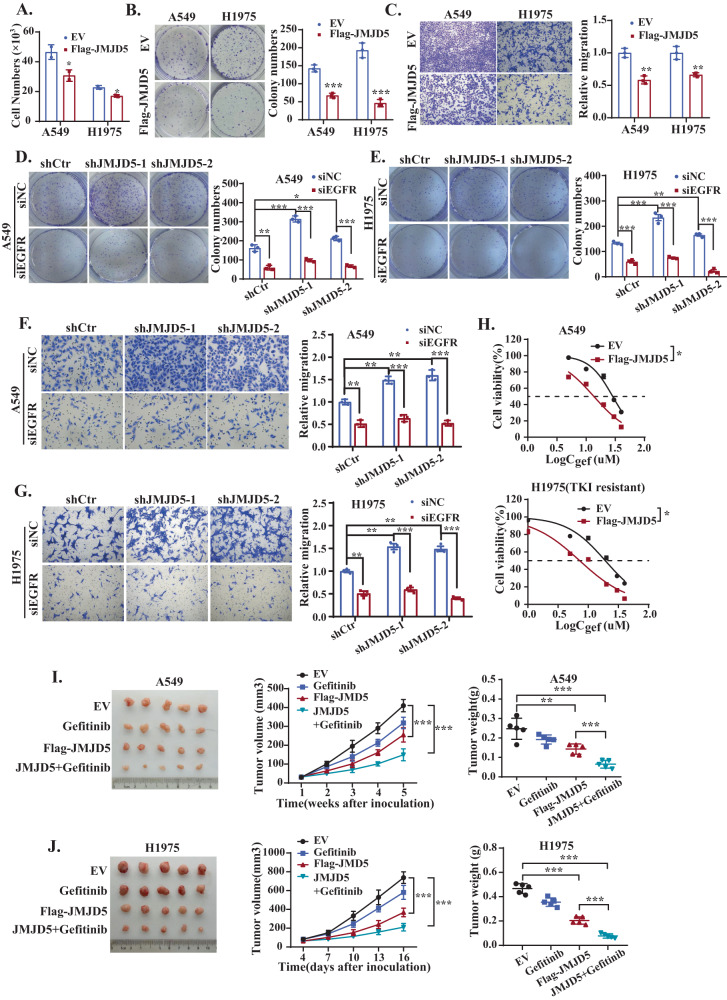
JMJD5 inhibits cell proliferation, migration and promotes gefitinib-sensitivity in lung cancer cells. Cell counting (**A**), colony formation (**B**), and transwell assay (**C**) analyses of the cell proliferation and migration in JMJD5 stably expressed A549 and H1975 cells. Colony formation (**D**, **E**) and transwell assay (**F**, **G**) analyses of JMJD5 silencing cells after EGFR siRNA treatment. **H** A549 or H1975 cells stably expressing JMJD5 were treated with an indicated dose of gefitinib for 48 h followed by MTT assays. The analyses were repeated three times, and the results were expressed as mean ± SD. *P < 0.05, **P < 0.01, ***P < 0.001. Xenograft tumor growth assays (*n* = 5/group) of A549 (**I**) and H1975 (**J**) cells after JMJD5 overexpression alone or in combination with gefitinib treatment. Photos of tumors and tumor growth curves were shown.

Our aforementioned results showed that JMJD5 negatively regulates EGFR stability, therefore, we further investigated the effects of JMJD5 on cell proliferation and tumor growth. The results showed that JMJD5 expression markedly suppressed cell proliferation (Fig. 6A, B) and migration (Fig. 6C) in A549 and H1975 cells, while silencing JMJD5 significantly promoted these events (Fig. 6D–G). Simultaneous depletion of EGFR effectively rescued JMJD5 knockdown-induced growth promotion, indicating that JMJD5 inhibits NSCLC cell proliferation and migration via downregulation of EGFR expression. Moreover, we also analyzed the effects of JMJD5 on the EGFR-TKI resistance in NSCLC. As shown in Fig. 6H, the IC_50_ assay showed that ectopic JMJD5 expression greatly reduced the resistance of EGFR-WT and EGFR-TKI-resistant mutant cell lines to gefitinib. In addition, JMJD5 overexpression combined with gefitinib significantly reduced EGFR expression and suppressed the growth of gefitinib-resistant NSCLC in xenograft mouse models (Fig. 6I, J, Fig. S5A, B). Together, these data support that JMJD5 inhibits cell growth and migration by reducing EGFR expression, thereby enhancing the sensitivity of NSCLC cells to gefitinib.

### Exosomal JMJD5 protein suppresses NSCLC growth as a potential therapeutic strategy

**Fig. 7:**
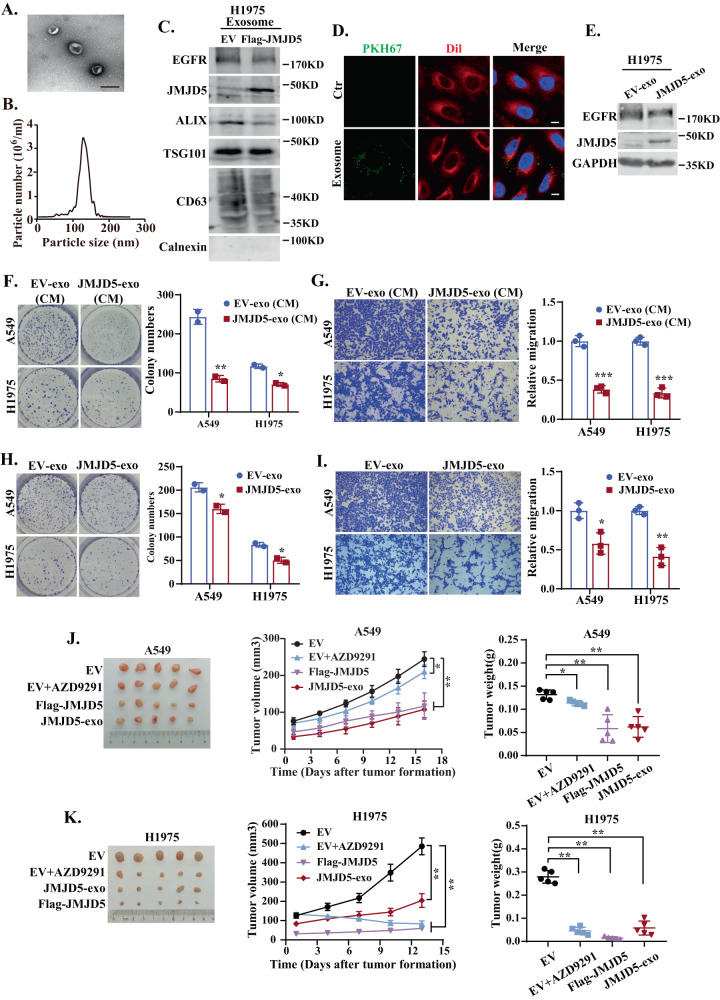
Exosomal JMJD5 protein suppresses NSCLC growth as a potential therapeutic strategy. Electron microscopy analysis (**A**) and nanoparticle tracking analysis (**B**) of exosomes isolated from H1975 cell culture supernatant. The scale bar represents 200 nm. **C** Western blotting analysis of exosome markers (ALIX, TSG101, CD63, calnexin), JMJD5, and EGFR protein levels in exosomes. **D** Confocal microscopy of H1975 cells (labeled with DiI, red) treated with exosomes (labeled with PKH67, green). The nucleus of cells was labeled with DAPI (blue). The scale bar represents 7.5 μm. **E** Western blotting analysis of the JMJD5 and EGFR protein levels of H1975 recipient cells after exosomes treatment. Colony formation (**F**, **H**) and transwell assay (**G**, **I**) analyses of A549 and H1975 cells after cultured with exosome-containing conditioned medium (CM) or isolated exosomes. The analyses were repeated three times, and the results were expressed as mean ± SD. *P < 0.05, **P < 0.01, ***P < 0.001. **J** Xenograft tumor growth assays (*n* = 5/group) of A549 (**J**) and H1975 (**K**) cells after AZD9291 treatment, JMJD5 overexpression or exo-JMJD5 treatment. Photos of tumors and tumor growth curves were shown.

We have previously reported that JMJD5 is downregulated in malignant effusions [33], however, its underlying secretion mechanism remains unclear. Since JMJD5 lacks a typical secretion signal, we analyzed the expression of JMJD5 in exosomes in the supernatant of lung cancer cells. As shown in Fig. 7A, B, electron microscopy showed that exosomes were round vesicles with intact membrane structures, and nanoparticle tracking analysis indicated the size of the exosomes to be in a range of 50–150 nm. Western blotting further confirmed the expression of exosome-positive marker ALIX, TSG101, CD63, and negative marker calnexin in our exosome preparations, indicating successful isolation of exosomes (Fig. 7C). The results showed that JMJD5 was expressed in exosomes derived from H1975 cells, and overexpression of JMJD5 could significantly increase its exosomal enrichment. Intriguingly, ectopic expression of JMJD5 also significantly inhibited EGFR expression in exosomes.

Then we investigated whether exosomal JMJD5 (exo-JMJD5) could be transferred to recipient cells. The PKH67-labeled exosomes were incubated with recipient cells, and the cellular uptake of exosomes and subsequent upregulation of JMJD5 were confirmed by fluorescence microscopy and western blotting (Fig. 7D, E). Next, we measured the biological function of exo-JMJD5 in NSCLC cell lines with WT or mutant EGFR. We treated A549 and H1975 cells with exosome-containing conditioned medium or isolated exosomes. As shown in Fig. 7F–I, colony formation and transwell assay analysis revealed that cell proliferation and cell migration were significantly decreased after treatment. We further examined the effects of exo-JMJD5 on xenograft tumor growth. The third-generation EGFR TKI AZD9291, which can overcome T790M-mediated TKI resistance, significantly suppressed the tumor growth in H1975 xenografts, but had a weak effect on A549 xenograft tumors. In contrast, exo-JMJD5 or JMJD5 overexpression inhibited the tumor growth of both xenografts (Fig. 7J, K). Altogether, these results support that exo-JMJD5 inhibits NSCLC growth and can be used as a potential therapeutic strategy.

## Discussion

Targeted EGFR therapeutics such as TKIs significantly relieve the development of mutant EGFR-driven NSCLC; however, the emergence of acquired resistance is a challenging issue for TKI therapies [6]. Meanwhile, WT-EGFR also performs an essential role in EGFR TKI resistance and NSCLC progression [7–9]. Therefore, it is of great importance to develop new strategies targeting both mutant EGFR and WT-EGFR to overcome therapeutic resistance. It has been reported that EGFR protein stability is tightly controlled through multisite ubiquitination and association with different E3 ubiquitin ligases [3, 37]. Upon EGF stimulation, E3 ligase c-Cbl catalyzes EGFR ubiquitination for subsequent lysosomal degradation [38], whereas F-box protein FBXL2 and E3 ligase CHIP target EGFR for proteasome-mediated degradation [13, 39]. In contrast, E3 ligases SMURF2 and WWP1 have been reported to interact with EGFR to enhance its ubiquitination and stability [12, 40]. In this study, we showed that JMJD5 cooperates with E3 ligase HUWE1 to destabilize EGFR and inhibit its signaling activity through proteasome degradation, not only for the WT-EGFR but also for the activating and gatekeeper mutants. Overexpression of JMJD5, therefore, significantly inhibits NSCLC cell growth and promotes the sensitivity of cells to EGFR TKI.

Unlike most other members of the JMJD family, which have additional DNA or protein-binding domains, JMJD5 contains the enzymatic JmjC domain only and belongs to the evolutionarily separated small JMJD subgroup which has more diverse functions other than regulating histone methylation [41]. Intriguingly, JMJD5 has been reported to influence important biological processes by promoting proteasomal degradation of key proteins, such as negatively regulating osteoclastogenesis through facilitating the degradation of transcription factor NFATC1 and modulating circadian rhythms via destabilizing the essential oscillator repressor CRY1 [15, 24]. In the current study, we demonstrate for the first time that JMJD5 acts as a crucial nexus to promote EGFR proteasomal degradation by recruiting E3 ligase HUWE1 and therefore also plays a critical role in tumorigenesis. Moreover, the interaction between JMJD5 and EGFR is not affected by EGFR mutations, either EGFR-activating mutations (L858R or 19del) or EGFR-TKI-resistant mutations (EGFR T790M or C797S). Our results support the notion that JMJD5 is a tumor suppressor in NSCLC through downregulating both WT and mutant EGFR, thereby inhibiting EGFR downstream signaling pathways and suppressing tumor growth. On the other hand, although our results revealed that there was no difference between the wild-type and the catalytically inactive mutant of JMJD5 in promoting EGFR ubiquitination, the JMJD5 mutant still had a slightly weaker effect on EGFR downregulation compared with the wild-type. Whether JMJD5 could regulate EGFR expression in both enzymatic activity-dependent and independent manner warrants further investigation.

The HECT-domain E3 ligase HUWE1 catalyzes ubiquitination of a diverse complement of key proteins such as p53, Myc, and Mcl-1, implicating that it is involved in the regulation of numerous cellular processes including cell proliferation, apoptosis, DNA repair, and stress response [42]. Since some HUWE1 substrates are oncoproteins and others are tumor suppressors, HUWE1 can be either oncogenic or tumor suppressing, mainly depending on the leading substrates it regulates in the context. Meanwhile, although HUWE1 has been reported to mediate EGFR ubiquitination and degradation in renal fibrosis inhibition [43], their association in cancer remains unclear. It has been reported that HUWE1 is overexpressed in lung cancer and promotes cell proliferation, migration, and invasion by targeting p53 and the RAC activator TIAM1 for proteasomal degradation [44, 45]. Our current results on the negative regulation of EGFR stability through the cooperation of HUWE1 and JMJD5 provide further insight into the complex role of HUWE1 in lung cancer progression. Since E3 ligase typically targets multiple substrates under different temporal and spatial conditions, its associated regulatory proteins may perform a critical role in determining the leading substrate and ultimate biological effects. Notably, although HUWE1 is upregulated in NSCLC tissues, EGFR protein levels are significantly reduced in patients with high JMJD5 expression and are correlated with better survival of the disease. These findings further support the importance of JMJD5 in regulating the HUWE1/EGFR axis.

Recent studies have found that exosomes or extracellular vehicles (EVs) are closely associated with cancer progression, diagnosis, and treatment [46, 47]. Due to their natural delivery ability, exosomes can be taken up by cells and stably transfer drugs, such as therapeutic miRNAs and proteins. Exosomal incorporation of tumor suppressors, such as miRNA let-7a and p53 protein, has been reported to promote cancer cell apoptosis and inhibit tumor development [48, 49]. In this study, we demonstrated that JMJD5 protein can be packaged into exosomes and delivered to the recipient cells to suppress NSCLC cell proliferation, migration, and xenograft tumor growth. Hence, it is likely that augmenting the expression level of JMJD5 in tumor cells through the uptake of exosomes, subsequently facilitating the degradation of intracellular EGFR, represents a novel tumor-suppressive mechanism of JMJD5. Moreover, our results showed that enhanced JMJD5 expression also significantly inhibits the exosomal enrichment of EGFR, likely due to its promotion of intracellular degradation of this protein. Since EGFR per se acts as a major cargo of EVs and contributes to tumor metastasis, angiogenesis, and TKI resistance [3], exosomal delivery of JMJD5 may represent a putative strategy for EGFR-targeted therapy of NSCLC.

In summary, our finding demonstrates that JMJD5 negatively regulates EGFR expression and signal activation by facilitating HUWE1-mediated EGFR degradation. Enhancing the expression and exosomal transferring of JMJD5 to accelerate EGFR degradation, therefore, is a potential therapeutic option for the treatment of TKI-resistant NSCLC.

### Supplementary information

Supplementary Information

Original Data File

reproducibility checklist

## Data Availability

The datasets used in this study are available from the corresponding authors upon reasonable request.
