# Supplementary material for: JMJD5 inhibits lung cancer progression by facilitating EGFR proteasomal degradation
Source: Cell Death Dis. 2023 Oct 9;14(10):657. doi: 10.1038/s41419-023-06194-0 (PMC10562424; doi:10.1038/s41419-023-06194-0)
Supplement: Supplementary file 1 — Supplementary Information [file 41419_2023_6194_MOESM1_ESM.pdf]

A.

UniProtKB	Name	Description of protein	Identified peptides
P00533	EGFR	Epidermal growth factor receptor	ITDFGLAK<br>YLVIQGDER<br>NLQEILHGAVR<br>ELVEPLTPSGEAPNQALLR
Q7Z6Z7	HUWE1	HECT, UBA And WWE Domain Containing E3 Ubiquitin Protein Ligase 1	SLLSILQR<br>IVNQPSSLFGSK<br>LGSSGLGSASSIQAAVR

B.

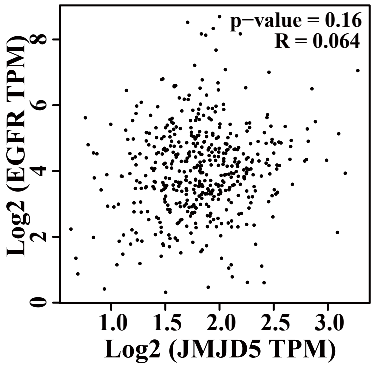

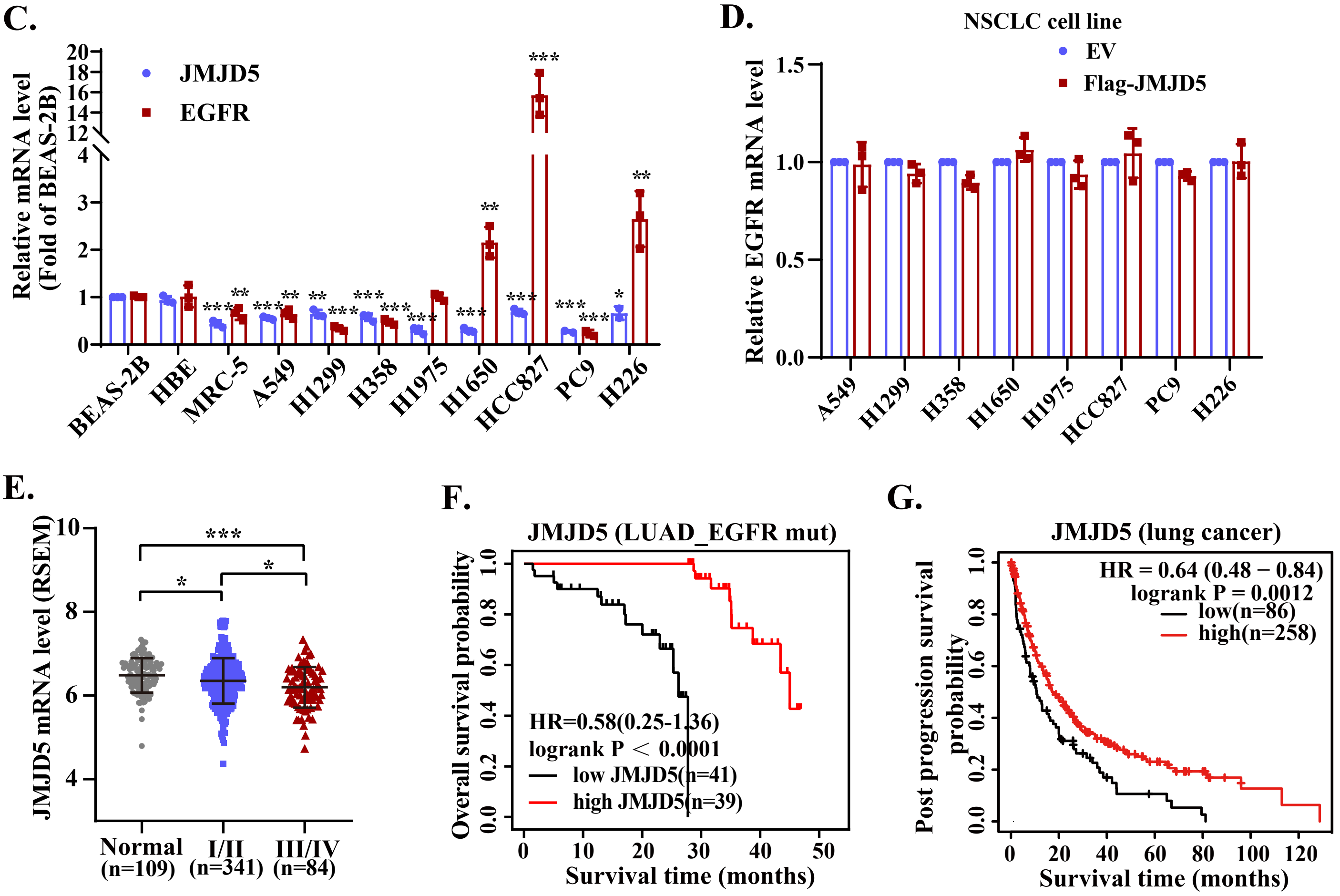

Supplementary Fig 1

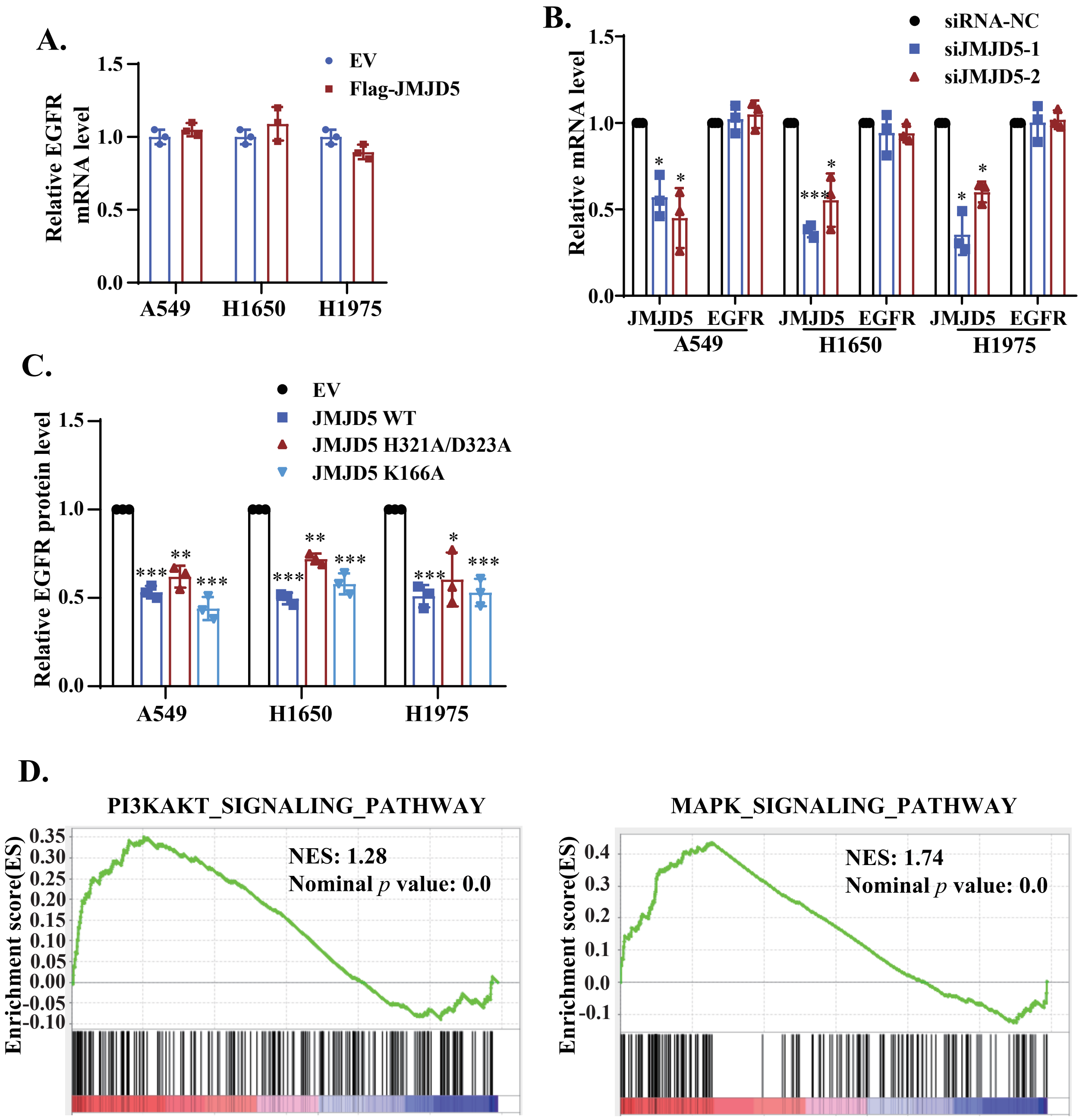
A.
Relative EGFR mRNA level
EV
Flag-JMJD5
A549
H1650
H1975
B.
Relative mRNA level
siRNA-NC
siJMJD5-1
siJMJD5-2
JMJD5 EGFR JMJD5 EGFR JMJD5 EGFR
A549
H1650
H1975
C.
Relative EGFR protein level
EV
JMJD5 WT
JMJD5 H321A/D323A
JMJD5 K166A
A549
H1650
H1975
D.
PI3KAKT_SIGNALING_PATHWAY
Enrichment score(ES)
NES: 1.28
Nominal p value: 0.0
MAPK_SIGNALING_PATHWAY
Enrichment score(ES)
NES: 1.74
Nominal p value: 0.0

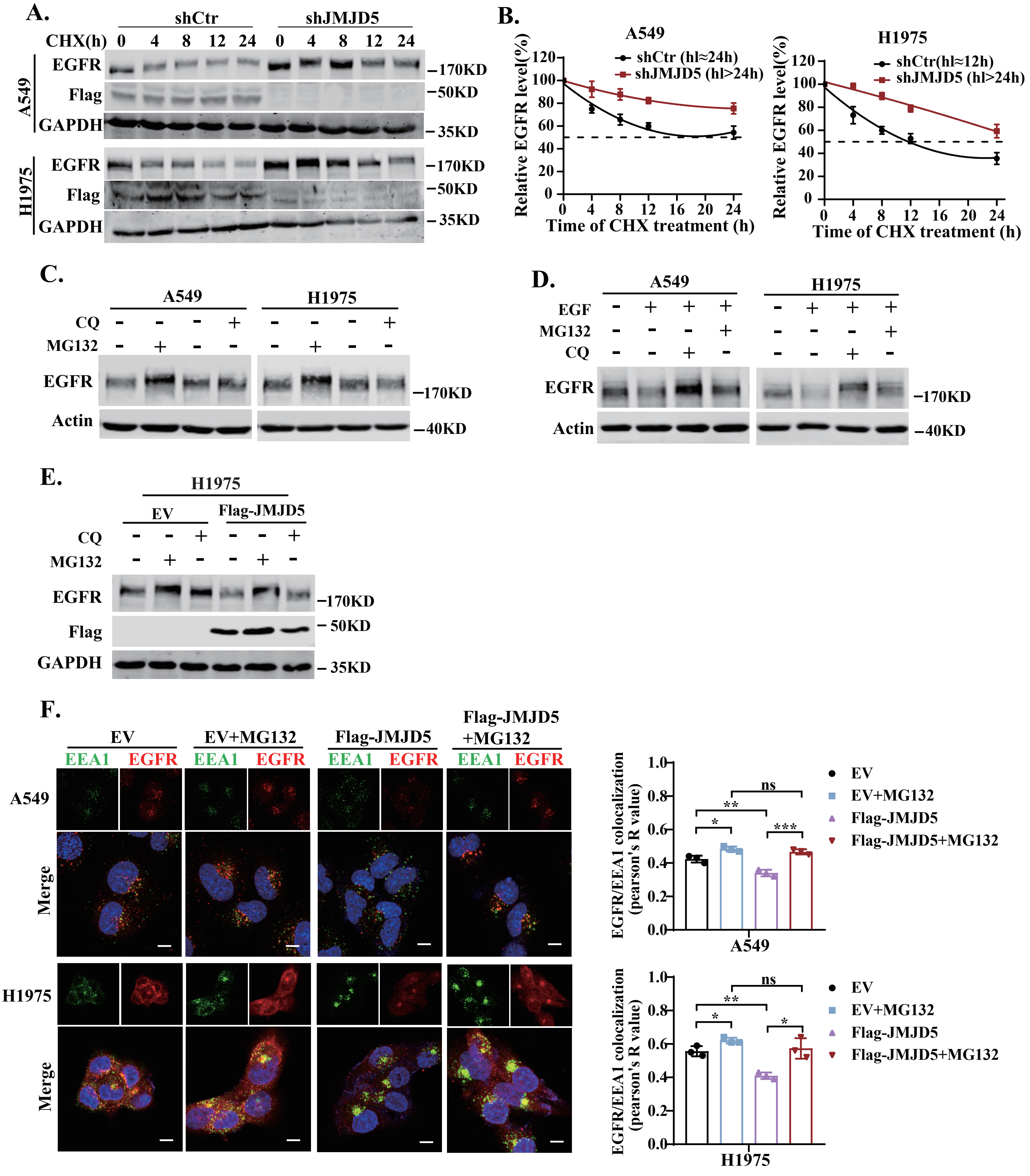

Supplementary Fig 3

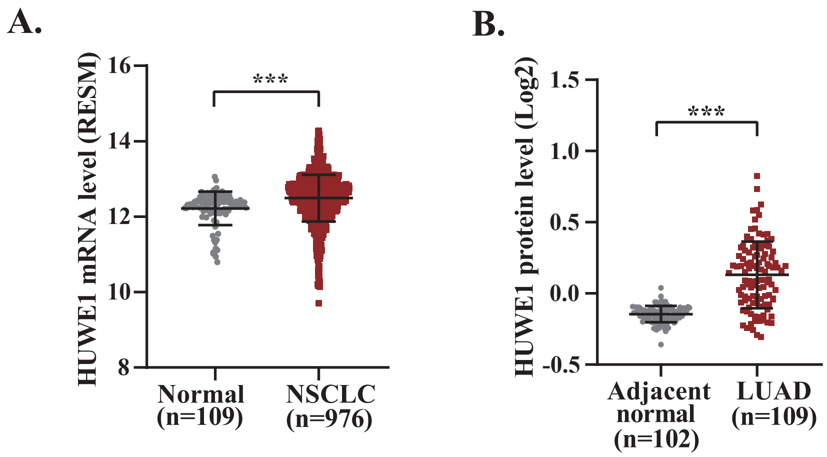

Supplementary Fig 4

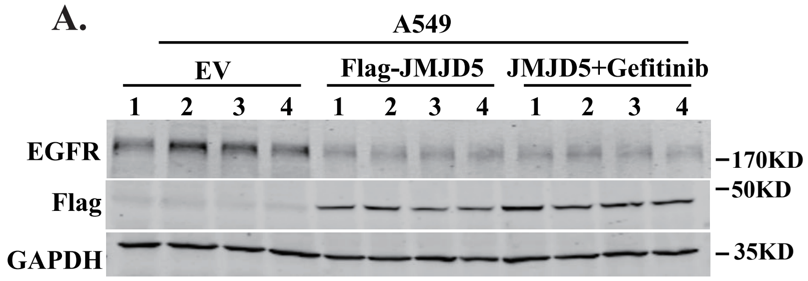

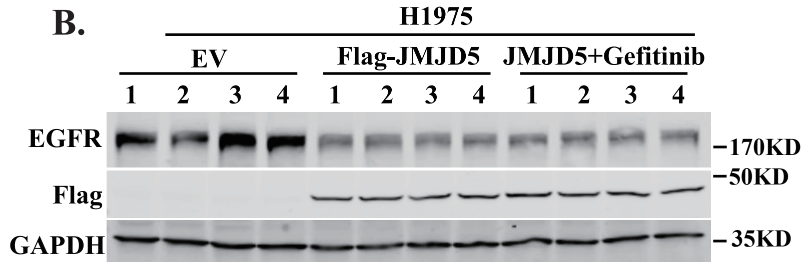

Supplementary Fig 5

Supplementary Figure Legends

Supplementary Fig 1. (A) Mass spectrometry analysis of JMJD5-associated proteins and the identified peptides of EGFR and HUWE1 were shown. (B) TCGA database analysis of the correlation between JMJD5 and EGFR mRNA expression in LUAD samples. (C) RT-qPCR analysis of JMJD5 and EGFR mRNA expression in human bronchial epithelial cells (BEAS-2B, HBE), human embryonic lung fibroblasts (MRC-5) and NSCLC cells (A549, H1299, H358, H1975, HCC827, H1650, PC9, and H226) (n=3). (D) RT-qPCR analysis of EGFR mRNA expression after JMJD5 overexpression. EV: empty vector. (E) TCGA database analysis of JMJD5 mRNA expression in LUAD tissues with different stages. Kaplan-Meier plots of overall survival (F) or post progression survival (G) of human lung cancer patients were stratified by JMJD5 levels from the Kaplan-Meier plotter database. *P < 0.05, **P < 0.01, ***P < 0.001.

Supplementary Fig 2. RT-qPCR analysis of EGFR mRNA levels in NSCLC cells after JMJD5 overexpression (A) or siRNA knockdown (B). (C) Quantitative analysis of EGFR protein levels detected by Western blot after ectopically expressing JMJD5 wild-type (WT) or mutants (H321A/D323A or K166A). The analyses were repeated three times, and the results were expressed as mean ± SD. *P < 0.05, **P < 0.01, ***P < 0.001. (D) Gene set enrichment plots of downregulated genes belonging to the PI3K/AKT and MAPK signaling pathway in JMJD5 stably expressed cells.

Supplementary Fig 3. (A-B) Control or JMJD5 silenced A549 or H1975 cells were treated with CHX (100 μg/ml) at indicated intervals, and protein stability of EGFR

was analyzed by Western blot. The analyses were repeated three times. (C-D) A549 or H1975 cells were treated with MG132 (10 μM, 6 h), CQ (20 μM, 6 h) or EGF (100 ng/ml, 1 h) before Western blot analyses. (E) H1975 cells stably expressing JMJD5 were treated with MG132 (10 μM, 6 h) or CQ (20 μM, 6 h) before Western blot analyses. (F) Colocalization analysis of EGFR with EEA1 in control and JMJD5 overexpressing A549 or H1975 cells after EGF (100 ng/ml, 30 min) and MG132 (10 μM, 6 h) treatment. The scale bar represents 7.5 μm. Quantification of EGFR/EEA1 colocalization was shown as Pearson's coefficient. ns: no significant, *$P < 0.05$, **$P < 0.01$, ***$P < 0.001$.

Supplementary Fig 4. (A) TCGA database analysis of HUWE1 mRNA expression in NSCLC and normal tissues. (B) CPTAC database analysis of HUWE1 protein expression in LUAD and adjacent normal tissues. *** $P < 0.001$.

Supplementary Fig 5. Western blot analysis of EGFR and Flag-JMJD5 protein expression in A549 (A) or H1975 (B) xenograft tumors with JMJD5 overexpression alone or in combination with gefitinib treatment.

Supplementary Table 1. siRNAs and shRNA

	sense	antisense
JMJD5 siRNA-1	GUGAUCCUGGGCUACUCCU	AGGAGUAGCCCAGGAUCAC
JMJD5 siRNA-2	GAAGUUGGUUCGAGGUACA	UGUACCUCGAACCAACUUC
EGFR siRNA-1	CGCAAAGUGUGUAACGGAAUA	UAUUCCGUUACACACUUUGCG
EGFR siRNA-2	GUGAGGUGGUCCUUGGGAA	UUCCCAAGGACCACCUCAC
HUWE1 siRNA-1	GAGUUUGGAGUUUGUGAAGUU	AACUUCACAAACUCCAAACUC
JMJD5 shRNA1	GATCCGCCACTGAGCTCTTCTACGACTCGAGTCGTAGAAGAGCTCAGTGGTTTTTG	AATTCAAAAACCACTGAGCTCTTCTACGACTCGAGTCGTAGAAGAGCTCAGTGGCG
JMJD5 shRNA2	GATCCGTCAACGAGTTCATCAGCAACTCGAGTTGCTGATGAACTCGTTGATTTTTACGCGTG	AATTCACGCGTAAAAATCAACGAGTTCATCAGCAACTCGAGTTGCTGATGAACTCGTTGACG

Supplementary Table 2. RT-qPCR primers

Gene	Forward Primer (5'-3')	Reverse Primer (5'-3')
JMJD5	CCATCAATGCCTGGTTTGGTC	GTGCGTGTCATGAGGGTACAGAG
EGFR	GTAACAAGCTCACGCAGTTGG	GTTGAGGGCAATGAGGACAT
GAPDH	ATGGGGAAGGTGAAGGTCGGAGT	TGACAAGCTTCCCGTTCTCAGCC
COL6A3	CCTAACCACATATGTTAGTGGAG	GAATGTCTCGCTTGCTCTCTG
FN1	GAGAATAAGCTGTACCATCGCAA	CGACCACATAGGAAGTCCCAG
F2R	GCTTCGGACCCACAAACG	CCTCTGTGGTGGAAGTGTGA
ITGA3	GCAGCCTCTTCGGCTACTC	CCGGTTGGTGTAGCCATC
LAMB1	AACTGGCAGGCAAGCTACAA	GGCGTTGTGTGCAACAGTAA
STMN1	ATTCTCAGCCCTCGGTCAAA	TCTCGTGCTCTCGTTTCTCA
VEGFA	GAGGGCAGAATCATCACGAAG	TGTGCTGTAGGAAGCTCATCTCTC
BDNF	ACCCATGGGATTGCACTTGG	AGCTGAGCGTGTGTGACAGT

MAPK3	CTAAGGAGCGGCTGAAGGAG	GCCTCAGCAAAGGAGAGAGG
MAP3K5	AAATCTTCATGGAGCAGGTC	ATTTGGAACGAAGGAGAGCA
