# Supplementary material for: JMJD5 inhibits lung cancer progression by facilitating EGFR proteasomal degradation
Source: Cell Death Dis. 2023 Oct 9;14(10):657. doi: 10.1038/s41419-023-06194-0 (PMC10562424; doi:10.1038/s41419-023-06194-0)
Supplement: Supplementary file 2 — Original Data File [file 41419_2023_6194_MOESM2_ESM.pptx]

## Slide 1
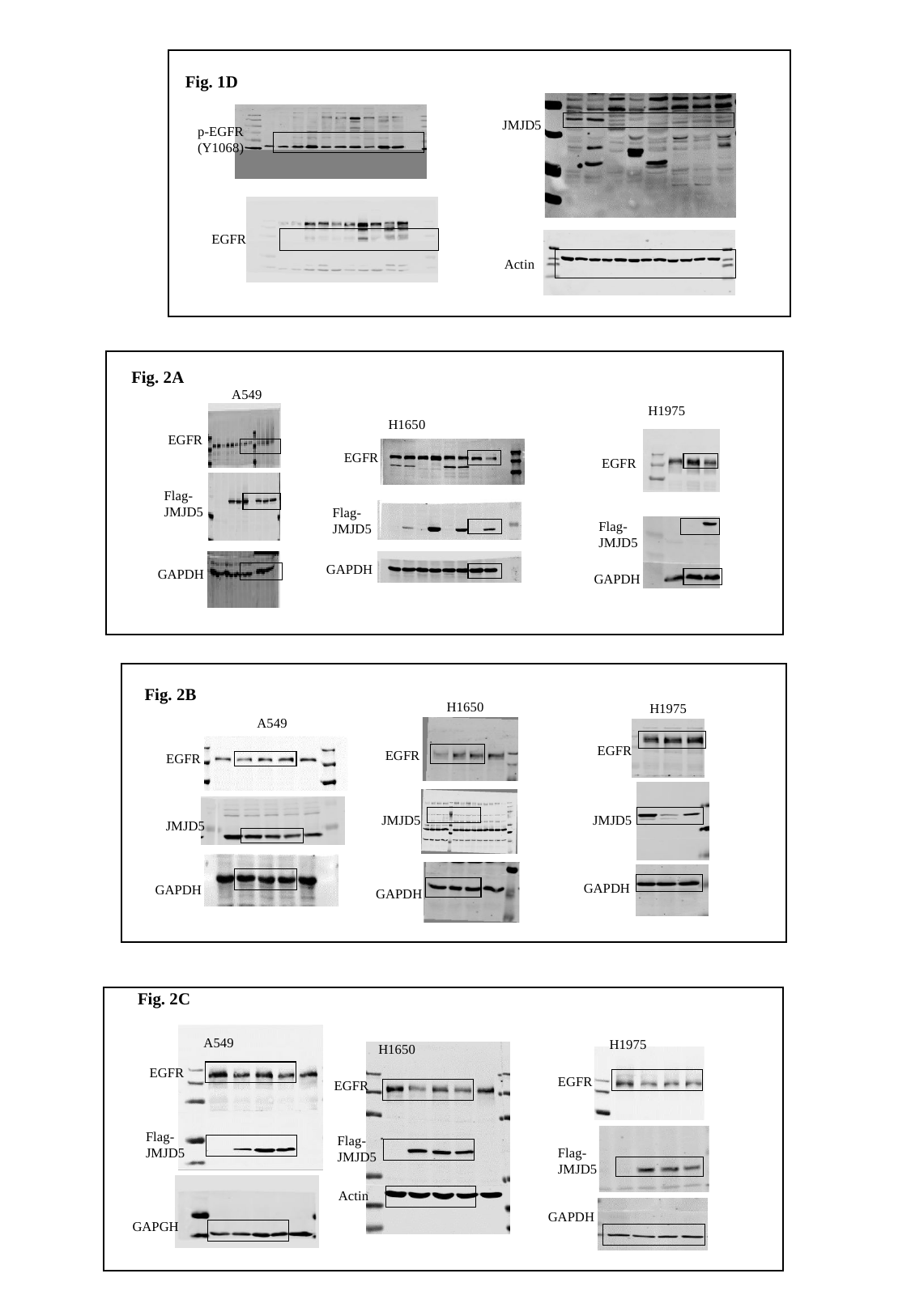

Fig. 1D
JMJD5
p-EGFR
(Y1068)
EGFR
Actin
Fig. 2A
A549
EGFR
Flag-JMJD5
GAPDH
H1975
EGFR
GAPDH
Flag-
JMJD5
H1650
EGFR
Flag-
JMJD5
GAPDH
Fig. 2B
H1650
EGFR
JMJD5
GAPDH
H1975
EGFR
JMJD5
GAPDH
A549
EGFR
JMJD5
GAPDH
Fig. 2C
A549
EGFR
Flag-
JMJD5
GAPGH
H1650
EGFR
Flag-
JMJD5
Actin
H1975
EGFR
Flag-
JMJD5
GAPDH

## Slide 2
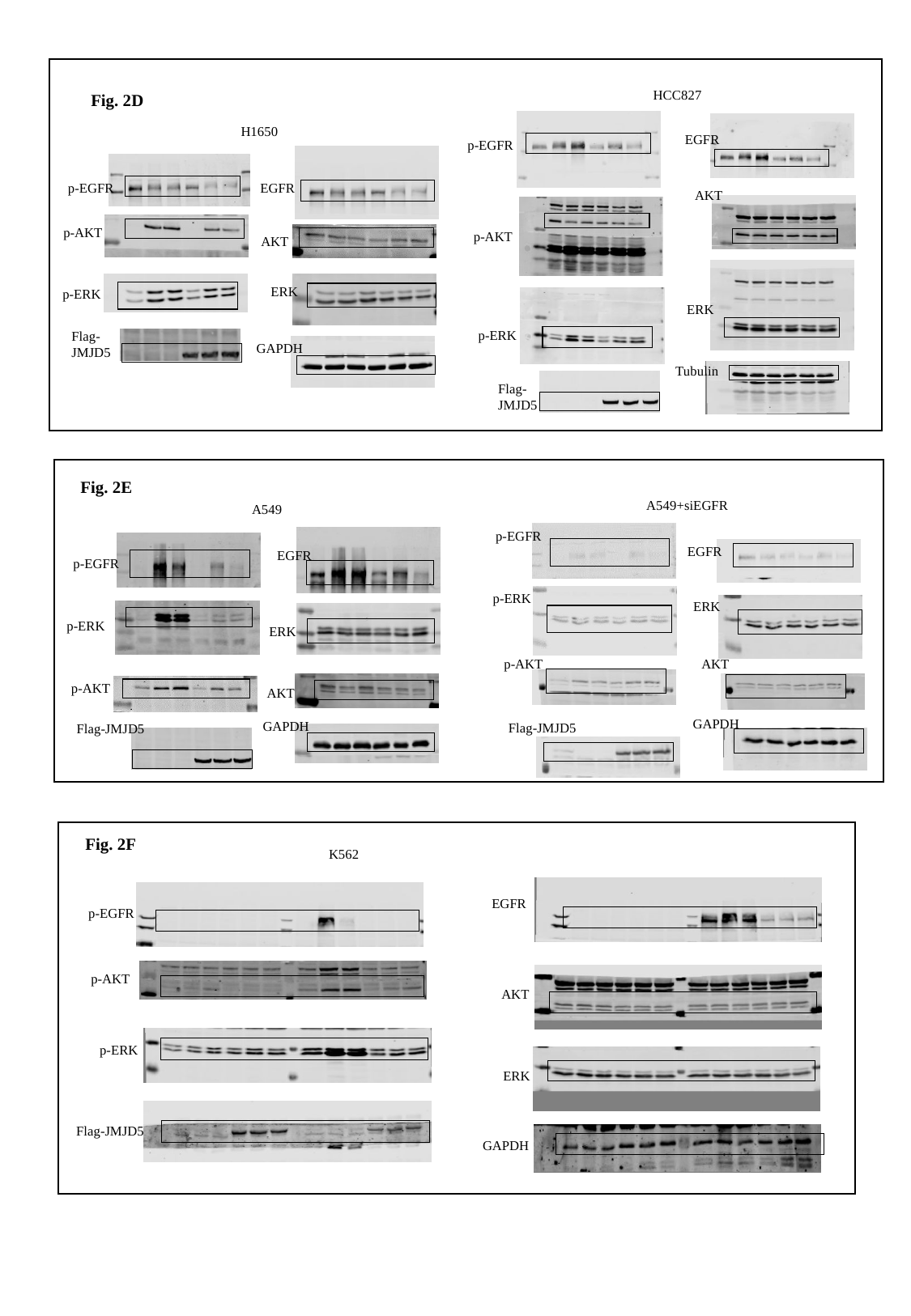

HCC827
p-EGFR
EGFR
AKT
p-AKT
ERK
p-ERK
Tubulin
Flag-JMJD5
Fig. 2D
H1650
EGFR
p-EGFR
p-AKT
AKT
ERK
p-ERK
Flag-JMJD5
GAPDH
Fig. 2E
A549+siEGFR
p-EGFR
EGFR
p-ERK
ERK
p-AKT
AKT
GAPDH
Flag-JMJD5
A549
EGFR
p-EGFR
p-ERK
ERK
p-AKT
AKT
GAPDH
Flag-JMJD5
Fig. 2F
K562
EGFR
p-EGFR
p-AKT
AKT
p-ERK
ERK
Flag-JMJD5
GAPDH

## Slide 3
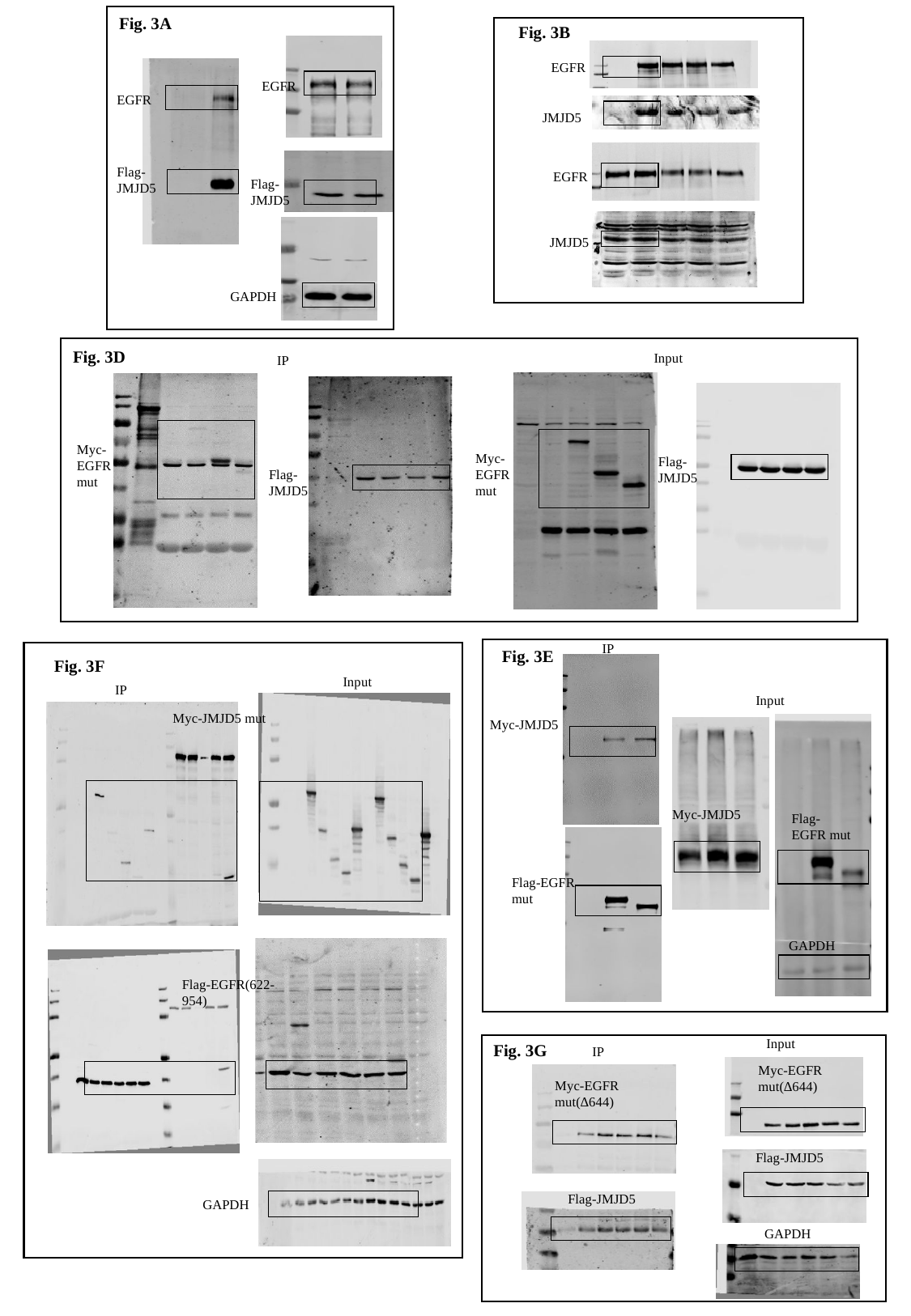

Fig. 3A
EGFR
EGFR
Flag-JMJD5
Flag-JMJD5
GAPDH
Fig. 3B
EGFR
JMJD5
EGFR
JMJD5
Fig. 3D
Myc-EGFR mut
Myc-EGFR mut
Flag-JMJD5
Flag-JMJD5
Input
IP
IP
Fig. 3E
Fig. 3F
Input
IP
Myc-JMJD5 mut
Flag-EGFR(622-954)
GAPDH
Myc-JMJD5
Input
Flag-EGFR mut
GAPDH
Myc-JMJD5
Flag-EGFR mut
Input
Fig. 3G
Myc-EGFR mut(∆644)
Myc-EGFR mut(∆644)
Flag-JMJD5
Flag-JMJD5
GAPDH
IP

## Slide 4
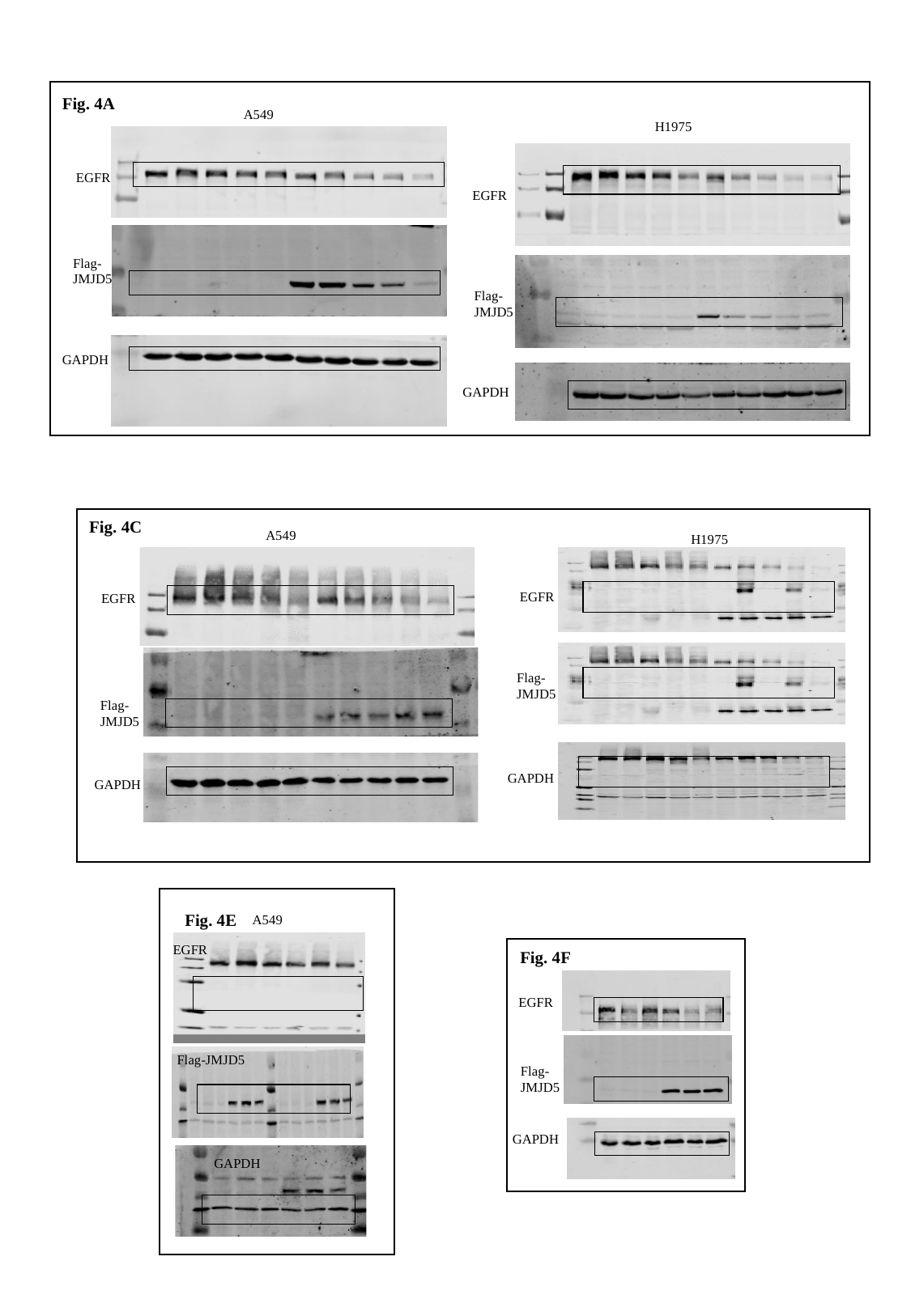

Fig. 4A
A549
EGFR
Flag-JMJD5
GAPDH
H1975
EGFR
Flag-JMJD5
GAPDH
Fig. 4C
A549
EGFR
Flag-JMJD5
GAPDH
H1975
EGFR
Flag-JMJD5
GAPDH
Fig. 4E
A549
EGFR
Flag-JMJD5
GAPDH
Fig. 4F
EGFR
Flag-JMJD5
GAPDH

## Slide 5
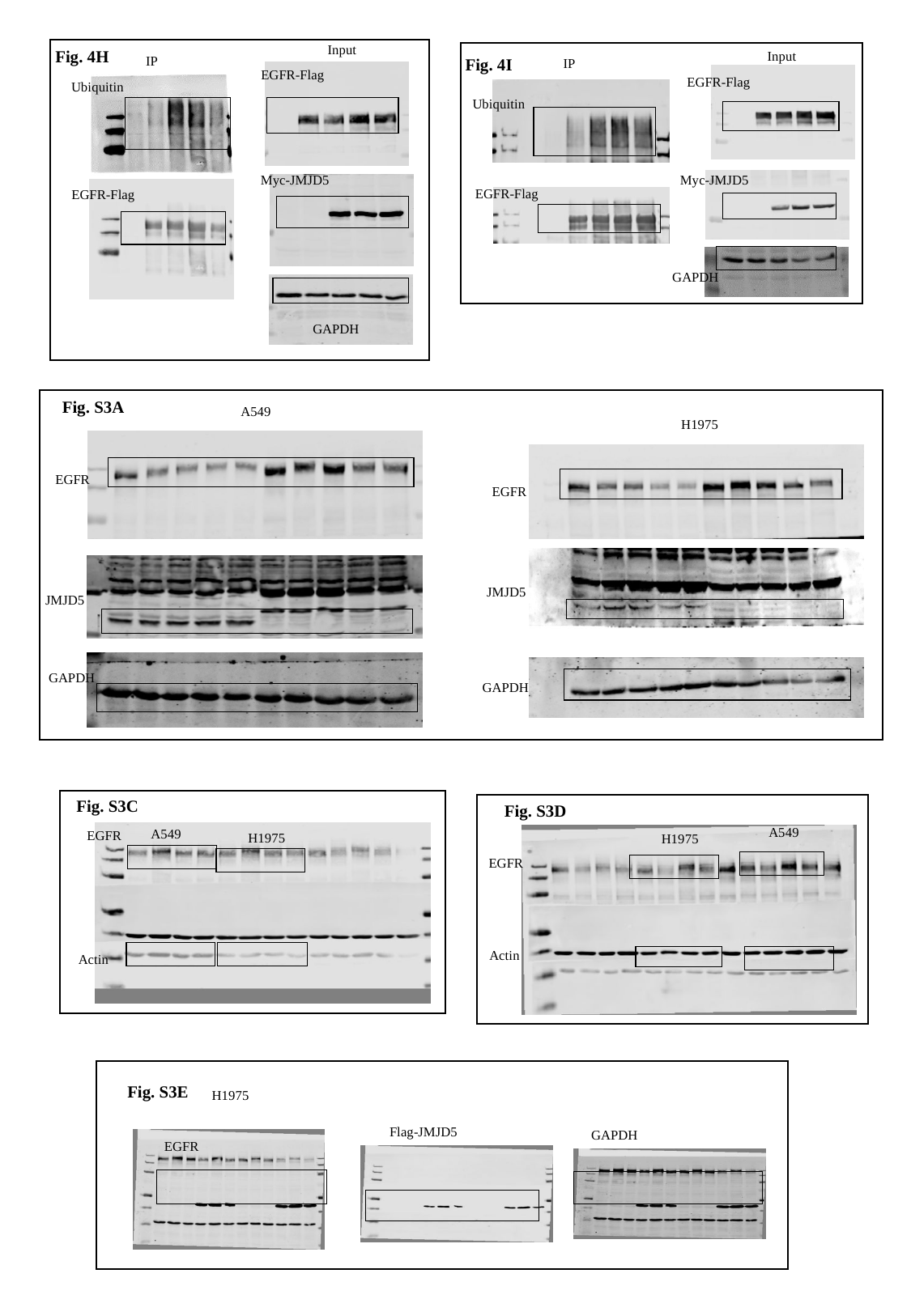

Input
Fig. 4H
EGFR-Flag
Ubiquitin
Myc-JMJD5
EGFR-Flag
GAPDH
Input
Fig. 4I
EGFR-Flag
Ubiquitin
Myc-JMJD5
EGFR-Flag
GAPDH
IP
IP
Fig. S3A
A549
EGFR
JMJD5
GAPDH
H1975
EGFR
JMJD5
GAPDH
Fig. S3C
A549
EGFR
H1975
Actin
Fig. S3D
A549
H1975
EGFR
Actin
Fig. S3E
H1975
Flag-JMJD5
GAPDH
EGFR

## Slide 6
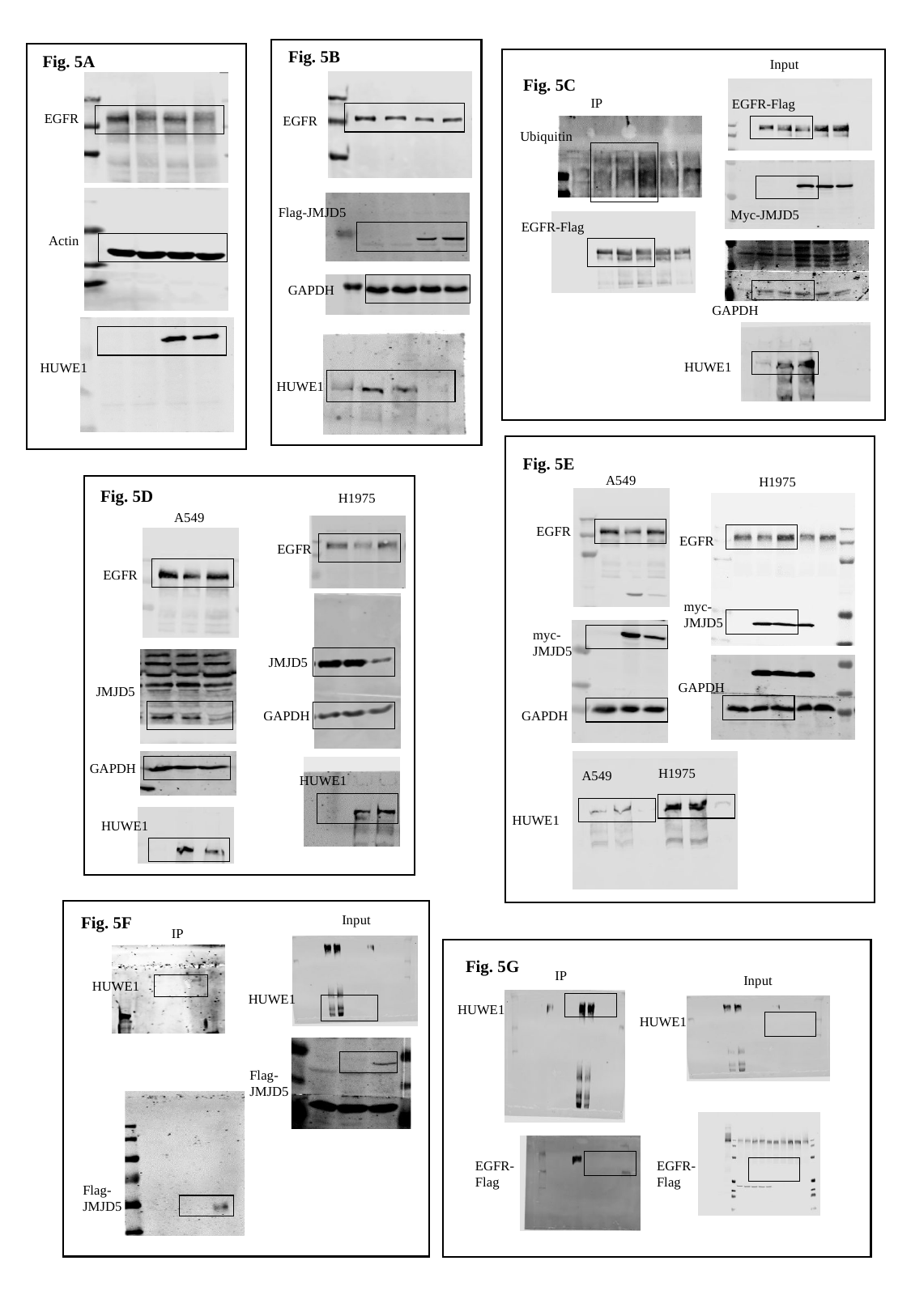

Fig. 5B
EGFR
Flag-JMJD5
GAPDH
HUWE1
Fig. 5A
EGFR
Actin
HUWE1
Fig. 5C
EGFR-Flag
Ubiquitin
Myc-JMJD5
EGFR-Flag
GAPDH
HUWE1
Input
IP
Fig. 5E
A549
H1975
EGFR
myc-JMJD5
GAPDH
EGFR
myc-JMJD5
GAPDH
H1975
A549
HUWE1
Fig. 5D
H1975
A549
EGFR
JMJD5
GAPDH
HUWE1
EGFR
JMJD5
GAPDH
HUWE1
Input
Fig. 5F
IP
HUWE1
HUWE1
Flag-JMJD5
Flag-JMJD5
Fig. 5G
IP
Input
HUWE1
HUWE1
EGFR-Flag
EGFR-Flag

## Slide 7
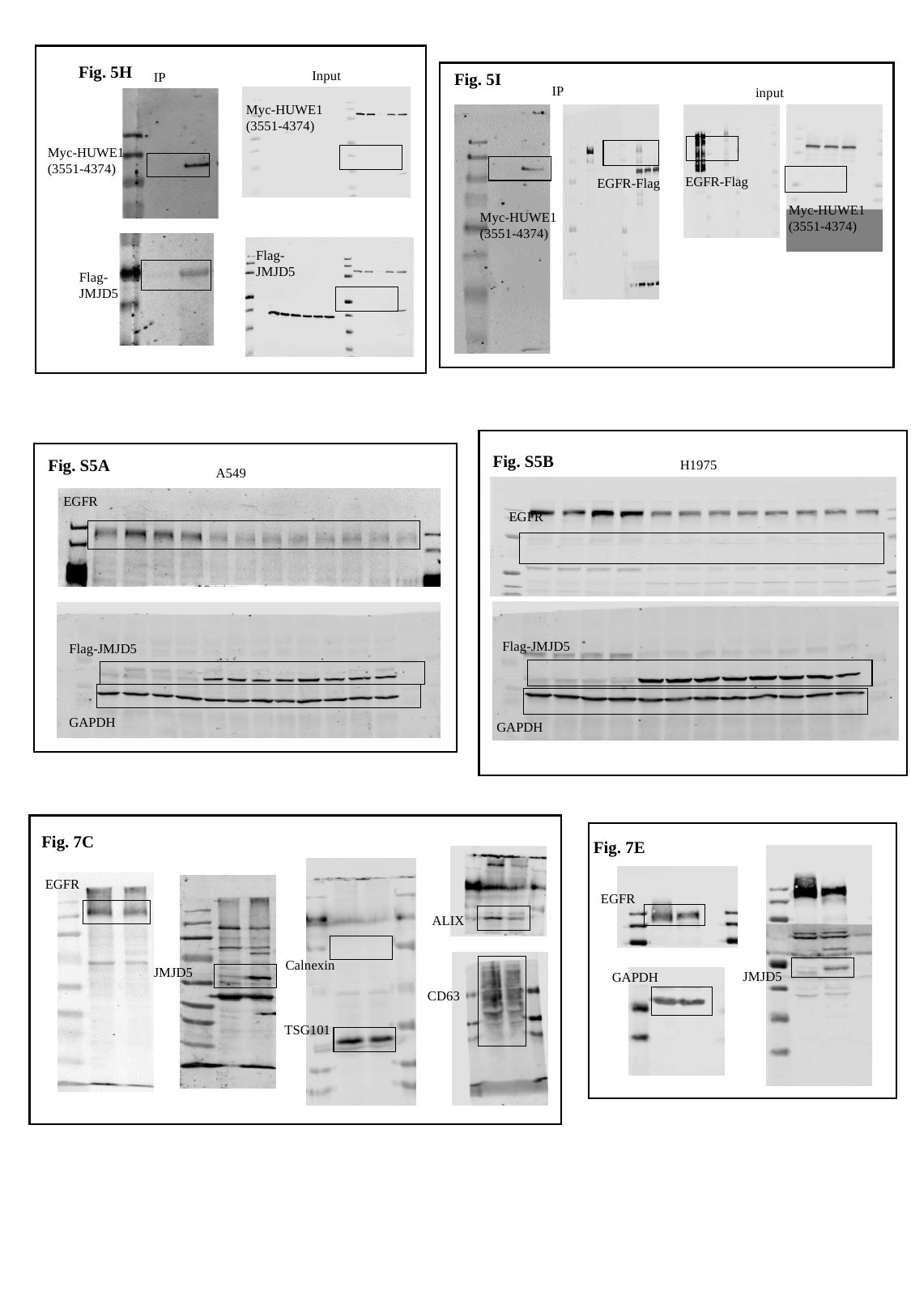

Fig. 5H
Input
Myc-HUWE1
(3551-4374)
Flag-
JMJD5
IP
Myc-HUWE1
(3551-4374)
Flag-
JMJD5
Fig. 5I
IP
input
EGFR-Flag
EGFR-Flag
Myc-HUWE1
(3551-4374)
Myc-HUWE1
(3551-4374)
Fig. S5B
H1975
EGFR
Flag-JMJD5
GAPDH
Fig. S5A
A549
EGFR
Flag-JMJD5
GAPDH
Fig. 7C
ALIX
Calnexin
TSG101
EGFR
JMJD5
CD63
Fig. 7E
JMJD5
EGFR
GAPDH
